# Constraints and advances in high-solids enzymatic hydrolysis of lignocellulosic biomass: a critical review

**DOI:** 10.1186/s13068-020-01697-w

**Published:** 2020-03-23

**Authors:** Ayla Sant’Ana da Silva, Roberta Pereira Espinheira, Ricardo Sposina Sobral Teixeira, Marcella Fernandes de Souza, Viridiana Ferreira-Leitão, Elba P. S. Bon

**Affiliations:** 1 Biocatalysis Laboratory, National Institute of Technology, Ministry of Science, Technology, Innovation and Communication, Rio de Janeiro, RJ 20081-312 Brazil; 2 Bioethanol Laboratory, Department of Biochemistry, Chemistry Institute, Federal University of Rio de Janeiro, Rio de Janeiro, RJ 21941-909 Brazil; 3 Laboratory of Analytical Chemistry and Applied Ecochemistry, Department of Green Chemistry and Technology, Ghent University, Coupure Links 653, 9000 Ghent, Belgium

**Keywords:** Lignocellulosic biomass, High solids loading, Enzymatic hydrolysis, Water constraint, Cellulases inhibition, Fed-batch strategy, Enzymatic hydrolysis reactors, Biomass sugar syrups, Biorefinery, Cellulosic ethanol

## Abstract

The industrial production of sugar syrups from lignocellulosic materials requires the conduction of the enzymatic hydrolysis step at high-solids loadings (i.e., with over 15% solids [w/w] in the reaction mixture). Such conditions result in sugar syrups with increased concentrations and in improvements in both capital and operational costs, making the process more economically feasible. However, this approach still poses several technical hindrances that impact the process efficiency, known as the “high-solids effect” (i.e., the decrease in glucan conversion yields as solids load increases). The purpose of this review was to present the findings on the main limitations and advances in high-solids enzymatic hydrolysis in an updated and comprehensive manner. The causes for the rheological limitations at the onset of the high-solids operation as well as those influencing the “high-solids effect” will be discussed. The subject of water constraint, which results in a highly viscous system and impairs mixing, and by extension, mass and heat transfer, will be analyzed under the perspective of the limitations imposed to the action of the cellulolytic enzymes. The “high-solids effect” will be further discussed vis-à-vis enzymes end-product inhibition and the inhibitory effect of compounds formed during the biomass pretreatment as well as the enzymes’ unproductive adsorption to lignin. This review also presents the scientific and technological advances being introduced to lessen high-solids hydrolysis hindrances, such as the development of more efficient enzyme formulations, biomass and enzyme feeding strategies, reactor and impeller designs as well as process strategies to alleviate the end-product inhibition. We surveyed the academic literature in the form of scientific papers as well as patents to showcase the efforts on technological development and industrial implementation of the use of lignocellulosic materials as renewable feedstocks. Using a critical approach, we expect that this review will aid in the identification of areas with higher demand for scientific and technological efforts.

## Background

The transition from an oil-based to a bio-based economy has been a continuing topic of discussion in the last few decades, largely due to its acceptance as one of the essential actions required to address climate change [1, 2]. In this context, the production of fuels and chemicals from lignocellulosic biomass (i.e., plant cell walls) has received extensive attention as this is the largest available renewable source of carbon, being the primary choice of feedstock for the development of biorefineries [3, 4].

The biochemical conversion of lignocellulosic biomass has been consolidated as the most appropriate method to depolymerize its polysaccharides into sugars, which are platform molecules that can be further converted into a wide array of fuels and chemicals through microbial fermentation or chemical processing [3, 5, 6]. Independently of the target products or the use of biochemical or chemical routes to process the biomass sugars syrups, the conversion of biomass includes two mandatory steps: pretreatment of the lignocellulosic biomass to render the material prone to the action of the enzymes, and the enzymatic hydrolysis of the pretreated material to produce sugar syrups [7, 8].

In the last few years, several industrial facilities that process biomass via enzymatic hydrolysis have been installed in different countries with the aim to produce cellulosic ethanol [5, 9–11]. However, many industrial units have reported difficulties in scaling up the pretreatment step, while the enzyme costs still significantly affect the final price of the target product, resulting in a negative impact on the technology’s competitiveness in the biofuels and chemicals market [5, 9]. Therefore, although the biochemical conversion of lignocellulosic biomass has reached considerable technical maturity, important technological advances are still needed for a significant contribution in a bioeconomy scenario.

One approach to improve the economic feasibility of the process is to increase the amount of biomass in the enzymatic hydrolysis reaction, which is referred to as “high-solids” enzymatic hydrolysis [12–14]. A process can be considered “high-solids” when the content of insoluble solids is such that no free water is present in the slurry at the onset of the hydrolysis reaction, which is generally observed at solids loadings higher than 15% (w/w) dry matter for most pretreated materials [15–17]. The use of high-solids content benefits the economics of lignocellulosic biomass conversion to fuels and chemicals by decreasing both capital and operational costs, as the increase in the final product concentration reduces equipment volumes alongside the costs for the separation steps, the water consumption, the wastewater generation, and the subsequent cost for disposal [18, 19]. In addition, this method also decreases the energy demand for the cooling and heating steps [13, 15]. Especially for processes aiming to produce cellulosic ethanol, the enzymatic hydrolysis with high solids loadings is of utmost importance [20] as it has been shown that the feasibility of the distillation step requires a fermentation broth with ethanol concentrations above 4% (w/w) [21, 22] and, by extension, an approximate concentration of 80–100 g/L of fermentable sugars in the biomass syrup.

However, high-solids enzymatic hydrolysis of insoluble lignocellulosic materials poses technical difficulties seemingly related to the reduced amount of free water. Under this condition, the fibrous material slurry has a high apparent viscosity that results in poor mixing and mass and heat transfer limitations, reducing the efficiency of the enzymes during the early stages of the hydrolysis, known as the “liquefaction stage” [23]. Alongside the solids increase, a decrease in the final glucose yield is also observed, which is generally known as the “high-solids effect” [24] and has been inconclusively attributed to water constraint [25]; to the inhibition of cellulolytic enzymes by the high concentration of its products, argued by many as the main factor contributing to the “high-solids effect” [24, 26]; to the increased concentration of sugar degradation products and hemicellulosic monosaccharides and oligosaccharides produced during the pretreatment step [26–29]; and to the unproductive binding of enzymes to lignin [30, 31].

The evaluation of the relative impact of these factors is challenging as the variables involved are interrelated, which hinders the individual assessment of the limiting factors. In addition, a stepwise evaluation intended to address the subject under comparative experimental conditions regarding the type of biomass, the pretreatment method, the enzyme formulation, the hydrolysis conditions, and the reactor design has not yet been conducted. This is a scenario prone to conflicting reports; while some have identified a prominent negative effect of a given parameter, others disagree, stating that the negative effect is equivalent regardless of the biomass loading.

Considering the importance of the subject to the economic competitiveness of the production of concentrated sugar syrups derived from biomass, this review has gathered relevant information in the area with the aim to provide a critical discussion on the constraints and advances in high-solids enzymatic hydrolysis of lignocellulosic biomass.

## Constraints in high-solids enzymatic hydrolysis

Many studies on the enzymatic hydrolysis of lignocellulosic biomass have been done using low and moderate solids loadings, which lessen the mixing difficulties posed by the presence of an insoluble substrate. Under these conditions, hydrolysis yields higher than 70% have often been reported depending on the type of biomass pretreatment and the cellulase loading [23, 28, 32–34]. Thus, the need to increase the glucose concentration in biomass sugar syrups, which is particularly driven by advances in the cellulosic ethanol industry, has compelled studies on high-solids enzymatic hydrolysis. However, under these conditions, a decrease in the hydrolysis rates and yields, known as the “high-solids effect,” has been observed; this effect is unfavorable to the efficiency of the saccharification process and leads to lower-than-expected glucose concentrations. Nevertheless, the reasons for the “high-solids effect” are not clearly understood. This section discusses the main factors that have been considered prominent causes of this effect.

### Effect of water constraint

Water is a key factor in the enzymatic hydrolysis of biomass polysaccharides, as it functions as a reactant in the cleavage of glycosidic bonds as well as the reaction medium that diffuses the enzymes, substrates, and sugars resulting from hydrolysis [25]. While there has been a significant amount of work on cellulose–enzyme interactions [35–37], the influence of water–biomass interactions and their effect on mass transfer during enzymatic hydrolysis have been overlooked. More recently, however, biomass–water interactions have been recognized as an important topic of investigation, particularly in high-solids enzymatic hydrolysis, as water can diffuse through the pores and bond to the plant cell wall matrix [38] in a phenomenon called water constraint [16, 25, 39]. Selig et al. [40] defined water constraint as the association of water molecules that are localized and structured, having limited degrees of freedom and kinetic motion when compared to bulk water.

Nuclear magnetic resonance (NMR) spectroscopy has been widely reported as a useful technique to measure the interaction of water with pure cellulose [25, 38, 40, 41] and with lignocellulosic biomass [16, 39, 42–48]. In high-solids enzymatic hydrolysis, this technique can be used to understand how water diffusion and constraint change as the solids content increases.

To assess information about biomass–water interactions, the NMR *T*_2_ (spin–spin) relaxation time of water’s hydrogen nuclei is used as an indicator of the extent to which water molecules are constrained within the biomass and also of their localization [49–51]. This approach is possible because *T*_2_ relaxation times will vary depending on free water availability, and faster relaxation times of the hydrogen nuclei will be observed for water pools that are constrained by tight interactions with the biomass in comparison to free water. The different pools of constrained water observed in studies of biomass–water interactions have been classified into several categories: primary bound water, which is related to the biomass surface interactions; secondary bound water, which is bound to water molecules already bound to the biomass; capillary water, which is bound within the cell wall lumens by capillary forces; and bulk or free water [16, 25, 38]. However, the number of water pools observed during high-solids enzymatic hydrolysis largely depends on the solids content and also on the type of biomass and pretreatment [48]. For example, three different water pools were observed in high-solids filter paper suspensions [38], while four different water pools of constrained water were identified in the evaluation of bacterial cellulose [25].

Felby et al. [38] developed one of the earliest studies on the use of NMR techniques to identify the enzyme–cellulose–water system in high-solids enzymatic hydrolysis using a 33% filter paper suspension as a model substrate. The authors showed that, in the early stages of hydrolysis (up to 4 h), the use of pure endoglucanases resulted in an increase in the relaxation time of the water pool associated with the cell wall. This was interpreted as the formation of cavities, called “enzymatic drilling,” that enabled the introduction of more water in the cellulose structure and increased the water-holding capacity; the use of cellobiohydrolase, however, had no effect on the water pools during the early stages of hydrolysis. When combining both endoglucanases and cellobiohydrolases, the synergistic effect of the enzymes produced the most pronounced effect on the localization of water, showing a significant change in the cell wall matrix. However, in that study, only one condition of the solids content was evaluated, which limited the discussion on the effect of water constraint and localization as solids increase.

Another study observed that an increase in the solids content, from 5 to 20% of bacterial cellulose, decreased the relaxation times of the less-constrained pools, meaning that those water pools became more closely bound to the biomass in higher solids loading conditions [25]. Interestingly, the relaxation time of primary bound water was constant, independently of the solids content, although the quantity of water in the primary bound water pool decreased as solids increased, demonstrating a decrease in the available surface area for water interaction. The correlation of the hydrolysis yields in 24 h and the relaxation times for different solids loadings suggested a relationship between increasing water constraints and decreasing hydrolysis efficiency.

In contrast, another study suggested that the water content was not an important factor that influences the decrease in yields for the hydrolysis of filter paper at high-solids content, based on experiments where various amounts of water were substituted by oleyl alcohol with no impact on yields; the investigation concluded that the inhibition of enzyme adsorption by hydrolysis products appeared to be the main cause for the observed solids effect [24]. In that study, however, the viscosity was kept constant, which could have masked the way the system would be affected in an actual high-solids condition.

Indeed, Roberts et al. [25] hypothesized that saccharification yields are impacted by the constraint of water due to mass transfer resistance, increased viscosity, and decreased diffusivities even before enzyme product inhibition can be observed. They found that soluble monosaccharides (glucose or mannose) created an additional water constraint, downshifting the relaxation times of the water pools and negatively affecting the hydrolysis efficiency. Therefore, as mannose is not an inhibitor of cellulases, the authors hypothesized that the solutes would impose an additional physical constraint on the water molecules interacting with the solutes, which would impact the enzymatic hydrolysis. Hence, a decrease in cellulose conversion yields was also observed as the concentration of glucose, mannose, galactose, xylose, or fructose increased [52]. However, as the impact on yields was different for the same concentration of different monosaccharides, the authors further investigated the correlation with the decrease in yields and the water constraint caused by each individual monosaccharide, finding a correlation coefficient of 0.94 for NMR *T*_2_ relaxation times and the glucose concentration produced in the presence of the monosaccharide.

In addition, increased concentrations of soluble species added to the hydrolysis reaction, such as sugar alcohols, which are commonly added as preservatives in commercial enzyme preparation, and free sugars, had a higher impact on the cellulose conversion yields than the increase of insoluble solids content [45]. To test this hypothesis, mixtures of 5% pure cellulose and increasing amounts of non-hydrolysable complex dextrans (Sephadex) were compared regarding their effect on water constraint and the effectiveness of the enzymatic hydrolysis. Even though the NMR measurements indicated that the cross-linked dextran was able to constrain water in a similar manner to cellulose, the increase in total insoluble solids by the addition of Sephadex in the reaction media resulted in a negligible difference in cellulose conversion. By comparing the water constraint and water availability, measured as water activity (*A*_w_), imposed by the insoluble solids and soluble compounds, the authors noted that, while insoluble solids had a more prominent effect on water constraint compared to the soluble species, soluble solids had a greater impact on water activity. Indeed, Liu and Chen [16] also compared the effect of adding cellulose, glucose, or xylose at different concentrations to steam-pretreated corn stover and determined that cellulose had by far the most pronounced effect for the constraint of water. Therefore, it was suggested that the decrease in yields during hydrolysis was more related to the decrease in water availability initiated by the increased concentration of all soluble species in high-solids conditions rather than by the increased water constraint associated with the increase in insoluble solids content.

Even with the demonstrated negative effects of the increase in soluble solids for the enzymatic hydrolysis, insoluble solids still have an impact on water availability and, therefore, on the yields obtained in high-solids conditions. The extent of this impact is, however, dependent on the composition of the insoluble phase. The relationship of water constraint and the inhibitory potential toward cellulose hydrolysis at low solids content was evaluated for a range of plant cell wall matrix polymers (hemicelluloses, pectins, and lignin), and it was concluded that insoluble hemicelluloses were responsible for the highest levels of water constraint and hydrolysis inhibition [40]. In addition, when comparing the effect of insoluble solids, Sephadex and Sigmacel 50 constrained water in a similar manner [45], while the much faster decline in the *T*_2_ relaxation curve of xylans indicated that they constrained water to a greater degree than cellulose [40], influencing the evaluation that each study had on the impact of insoluble solids content on water constraint.

More intriguing, and opposed to what was reported by Roberts et al. [25], a decrease, rather than an increase, in constrained water was seen when introducing soluble solids to 30% solids loadings enzymatic hydrolysis, even though yields did decrease [45]. The authors suggested that a shift in the distribution of water away from insoluble surfaces was responsible for the decreased yields. In agreement with those data, Liu and Chen [16] reported that the water *T*_2_ relaxation times for a mixture of steam-pretreated corn stover (12.5% solids) with 2–15% of glucose or xylose barely changed the profile of constrained water pools with the increase of solute concentration. One explanation for the effect that the solutes could impose on the system is related to the fact that the water that interacts with soluble species, despite having greater degrees of freedom than water constrained by insoluble solids, is less available for other uses [45]. Therefore, it was proposed that water constraint may not be as problematic as thought at high-solids loadings when compared to the lower availability of water on biomass, which may reduce the effectiveness of enzymes to access these surfaces.

In a subsequent study, the correlation between the effectiveness of enzymatic hydrolysis and the water constraint effect caused by cellulose I_β_, II, and III_I_ was assessed and further supported the hypothesis that surfaces with the highest water constraint are the most efficiently hydrolyzed [41]. In addition, stronger cell wall interactions with water were detected in agave samples when compared to switchgrass and poplar, also indicating that the water constraint by the agave biomass was correlated to its higher reactivity and facilitated hydrolysis [43]. Adding more support to this hypothesis, Weiss et al. [48] compared different pretreatments of wheat straw and spruce at high-solids hydrolysis and concluded that the highest yields were achieved with biomass samples that had more water in highly constrained pools at high solids loadings. In addition, this study showed that the most drastic decrease in enzymatic hydrolysis yields occurred with the disappearance of free water from the system, which resulted in a decrease in diffusion rates, and consequently, less-efficient diffusion of enzymes to the substrate. Therefore, the authors suggested that the high-solids effect seen was primarily a function of biomass–water interactions, both through water constraint and diffusion in the biomass matrix.

In a different approach, Liu and Chen [39] evaluated the distribution of water not only at the beginning of the hydrolysis but also along the saccharification process at different solids loadings of steam-exploded corn stover. They observed that the *T*_2_ relaxation times of all water pools became shorter with solids loadings increasing from 1 to 30%, which suggested stronger interactions between water and the biomass by the transformation of capillary water into bound water at higher solids loading, resulting in reduced water mobility. During the enzymatic hydrolysis, most of the constrained water was released before 36 h; however, as solids loadings increased, the release of constrained water was observed only after 48 or 96 h, which was consistent with the lower glucan conversion yields at higher solids content.

In summary, the aforementioned studies highlighted the critical importance of water in high-solids enzymatic hydrolysis in spite of some conflicting conclusions. Many studies indicated that water constraint was a key factor that negatively affected high-solids enzymatic hydrolysis, whereas others suggested that the hindered mobility of free-flowing water imposed by soluble species could be more problematic than the constraint of water by the insoluble biomass itself. For instance, the comparison of different substrates at high-solids enzymatic hydrolysis indicates that the ones capable of constraining more water had more favorable hydrolysis. As the evaluation of the effect of water constraint in high-solids enzymatic hydrolysis is relatively new, more in-depth studies with targeted experimental designs could provide new insights into the mechanisms and impact of water constraint for the “high-solids effect”. In addition, the closer evaluation of the interactions between water–biomass, water-soluble molecules, and water-insoluble polymers in high-solids enzymatic hydrolysis could help answer the open question about the real contribution of enzyme feedback inhibition in this system, which has been argued in many studies to be the main factor contributing to the “high-solids effect”. Although the influence of water–biomass interactions for the “high-solids effect” has been neglected in many studies, the development of new and more advanced techniques to measure the impact of water constraint will help researchers design experimental strategies and may be of key importance to develop efficient biomass conversion processes.

### Effect of inhibitors on enzyme efficiency

#### Effect of sugars and degradation products derived from biomass on enzyme efficiency

Cellulases, β-glucosidase, and hemicellulases end-product inhibition have been the subject of numerous studies [24, 26, 52–57]. Cellobiose directly inhibits both cellobiohydrolases and endoglucanases [54, 55, 57] by its binding to the catalytic [54] and/or carbohydrate-binding module [58], while glucose inhibits mainly β-glucosidase, and to a lesser extent, cellobiohydrolases and endoglucanases via its binding to the catalytic and/or to the carbohydrate-binding module of these enzymes [53, 55, 58]. Moreover, hemicellulose-derived monosaccharides, xylobiose, short xylooligosaccharides, and xylans, have also been shown to hinder the action of cellulases [27–29, 52, 53, 59–63] likely via an adsorption inhibitory mechanism that prevents the access of cellulases to the cellulose chain [24, 63]. These hemicellulose-derived sugars can result from the action of hemicellulases during enzymatic hydrolysis or derive from the breakdown of the hemicellulose chains during certain pretreatments, such as hydrothermal methods [64]. Other compounds resulting from the biomass pretreatment (i.e., furan derivatives and phenolic compounds) can also hamper the activity of these enzymes [26, 27, 60, 65–71].

The above-described phenomena would be intensified in a high-solids context and may significantly affect the hydrolysis rate and final yield obtained due to a reduction in the enzymatic activity. In fact, the exogenous addition of glucose, in the range of 60 to 200 g/L to mimic the concentrations achieved in high-solids loadings, resulted in an inhibitory effect higher than 20% regardless of the biomass, the pretreatment type, and the enzyme preparations [53, 56, 68, 72]. For instance, in the hydrolysis of 10% Avicel by Celluclast^®^ plus Novozyme^®^ 188, up to 70% cellulase inhibition was observed within 30 min of hydrolysis in response to the addition of 100 g/L glucose [53]. Silva et al. [56] reported that the presence of 60 g/L glucose at the onset of the enzymatic hydrolysis of 5% (w/w) hydrothermally treated sugarcane bagasse decreased the glucose yields in the range of 42–60% depending on the enzyme preparation used (Celluclast^®^ 1.5L plus Novozyme^®^ 188, Cellic^®^ CTec2 and a laboratory-made formulation).

Most of the studies reported in the literature were conducted with solids loading lower than 5%, where the target inhibitors were exogenously added [52, 54–56, 60–63, 65, 66, 68, 70, 73]. In high-solids hydrolysis, the lack of free water would hinder the appropriate diffusion of end-sugar products away from the reaction zone and active sites of enzymes, possibly leading to an even greater inhibitory effect [25, 52]. However, it is difficult to extrapolate results obtained with low-solids loadings to high-solids conditions, particularly because studies varying the solids loadings usually maintain the enzyme dosage constant per gram of substrate. Therefore, the ratio between the exogenously added compound and the enzyme concentration may be much higher at low-solids conditions, and consequently, a stronger inhibitory effect would be expected. In fact, few studies have reported the exogenous addition of inhibitors in enzymatic hydrolysis experiments with solids loadings higher than 15% and these have mostly found a lower reduction in yield than the previously mentioned studies [24–26, 67]. The addition of 50 g/L of glucose to a reaction medium with 20% filter paper resulted in a 12.3% lower glucose concentration after 48 h of hydrolysis [24]. In another study, a full factorial experimental design was used to evaluate not only the exogenous addition of monomeric sugars (124.9 g/L) but also of other possibly inhibitory compounds in the hydrolysis of 15% acid-pretreated corn stover [26]. These authors reported that the exogenous addition of glucose was responsible for a maximum decrease in cellulose conversion of 20%.

Even though cellobiose is a stronger inhibitor of cellulases than glucose [54], most studies, as the ones cited above, deal only with the effect of glucose accumulation under high-solids conditions. Although some researchers have reported the accumulation of cellobiose during the enzymatic hydrolysis at high-solids loadings [26, 72, 74], this accumulation could be mitigated by the addition of higher β-glucosidase loading. Indeed, many studies have reported that the use of enzyme formulations containing high β-glucosidase activity lessens or even avoids cellobiose accumulation [24, 25, 56, 68, 75, 76]. The inhibition by glucose, however, is much more challenging as this sugar is the end product of the enzymatic hydrolysis of cellulose and accumulates in the common batch configuration, being more attention-worthy.

Moreover, short xylooligosaccharides are postulated to be more powerful inhibitors than equal molar amounts of cellobiose and glucose under low-solids loadings [28, 59, 64]. The study of Kumar and Wyman [28] was the first report to show that xylobiose and higher xylooligosaccharides inhibited enzymatic hydrolysis of pure glucan, pure xylan, and pretreated corn stover. Qing et al. [59] reported that the presence of 12 g/L of xylooligosaccharides lowered the initial rate of hydrolysis of Avicel by 82%, while the final glucose yield decreased by 38%. Indeed, low DP xylooligosaccharides were recalcitrant toward several novel commercial enzyme blends (Cellic^®^ CTec2, Cellic^®^ HTec2, and Multifect^®^ pectinase) [62]. Although most studies suggest a substrate adsorption inhibitory mechanism that prevents cellulases from accessing the cellulose chain, Kont et al. [64] suggested that xylooligosaccharides with a degree of polymerization ranging from 7 to 16 might mimic the structure of the cellulose chain and bind to the active site of cellobiohydrolases. Nevertheless, no reports have investigated short xylooligosaccharides and xylobiose by adding these compounds in hydrolysis performed at high-solids conditions, so future investigations should also address these species and further clarify their inhibitory properties.

Another class of hydrolysis end-products that deserves attention is the oxidized sugars, such as cellobio-δ-lactone/cellobionic acid and glucono-δ-lactone/gluconic acid, that derive, respectively, from the action of cellobiohydrolases and β-glucosidase on oxidized cellodextrins. These oxidized cellodextrins result from the action of lytic polysaccharide monooxygenases (LPMOs), present in cellulase blends with improved catalytic efficiency (e.g., Cellic^®^ CTec2 and 3). The LPMOs and the hydrolytic enzymes show a synergistic action in cellulose degradation, where the former can account for approximately 5% of the glycosidic bonds cleavage by oxidation [77–81]. Few studies have documented the inhibitory effect of cellobio-δ-lactone and cellobionic acid on cellobiohydrolase I and cellobiose dehydrogenase activities [82], and d-glucono-1,5-lactone and gluconic acid on β-glucosidase activity [77, 78, 81]. Hildebrand et al. [82] reported that cellobionic acid was less inhibitory to cellobiohydrolase I than cellobiose, while Cannella et al. [77] observed a 50% β-glucosidase inhibition in the presence of 6 g/L of gluconic acid. Further studies are needed to understand the extent of inhibitions caused by such oxidized products in high-solids conditions.

Even though the presence of increasing concentrations of sugars has been shown to have an inhibitory effect on enzyme activity, Kristensen et al. [24], based on previous reports on inhibition of enzyme adsorption [58], evaluated if the increased sugar concentration at high solids would impact this phenomenon. As result, the authors suggested that the “high-solids effect” was mainly accounted by the inhibition of the efficient adsorption of cellulase to cellulose caused by the hydrolysis products, as only 17% of the added enzyme was adsorbed at 30% solids content, while 40% was adsorbed at 5% solids content. These authors also showed a statistically significant correlation between the decrease in conversion and the decrease in enzyme adsorption. Moreover, considering the water constraint perspective presented in the previous topic, it is suggested that, in parallel to the classical enzyme end-product inhibition, soluble sugars can affect enzymatic hydrolysis by increasing water constraint and/or decreasing water activity in cellulose suspensions even before impacting the enzyme activity [25].

Besides sugars, other inhibitory soluble compounds, such as furan derivatives resulting from sugar degradation and phenols, may be present at significant concentrations in the reaction mixtures of the enzymatic hydrolysis at high-solids loadings [26, 27]. These compounds can be generated in different amounts during the biomass pretreatment step, depending on the biomass type and pretreatment severity [26, 27, 60, 69]. In studies conducted using low solids, the phenolic compounds resulting from the degradation of lignin or released from the biomass as aromatic extractives have been reported as strong inhibitors of the cellulolytic system [26, 27, 60, 66, 69, 71]. Kim et al. [60] reported that phenols affected the cellulolytic system of Spezyme^®^ CP/Novozyme^®^ 188 even at low concentrations (0.3–3.5 g/L) and decreased the initial hydrolysis rate and sugar yield of 1% Solka Floc by 15% and 20%, respectively, in the presence of 3.5 g/L of phenolic compounds. In addition, Kim et al. [60] and Qin et al. [71] reported not only the deactivation of cellulases but also the co-precipitation of these enzymes and phenolic compounds, which, in part, explain their detrimental effect in the cellulolytic system. Polymeric phenols were also reported to exhibit a stronger inhibitory effect on cellulases when compared to monomeric phenols. The presence of 1 mM tannic acid decreased the initial rate and yields of the hydrolysis of 2.5% dilute acid-pretreated corn stover by 70–80% [60, 83]. These authors suggested that oligomeric phenols could inactivate cellulases by reversibly complexing with them or by adsorbing onto cellulose, hindering the cellulases’ productive binding. Xylanase activity was also reported to be affected by phenols (0–100 mM) [84].

Other authors argue against a significant inhibitory effect by phenols. Hodge et al. [26] reported that the enzymatic hydrolysis of 15% corn stover pretreated with dilute acid was only slightly inhibited in the presence of 9 g/L of phenolic compounds, 15 g/L of acetic acid, and 8 g/L of furans. A detailed investigation done by Du et al. [68] evaluated the hydrolysis of filter paper, Avicel, and carboximethycellulose (CMC) at 1% (w/w) in the presence of furfural (1–10 g/L), 5-hydromethylfurfural (5-HMF, 0.8–8 g/L), phenol (0.03–0.15 g/L), vanillin (0.05–1 g/L), formic acid (1–10 g/L), and acetic acid (1.5–15 g/L), and showed that phenolic compounds did not affect the hydrolysis carried out by Cellic^®^ CTec2. However, they reported a significant inhibition of the enzymatic hydrolysis of Avicel and CMC by furfural (1–10 g/L) and a stronger inhibition of filter paper hydrolysis by 5-HMF (0.8–8 g/L). A different observation was made by Kim et al. [60], who reported that furan derivatives and carboxylic acids did not affect the hydrolysis of 1% Solka-Floc, and by Di Risio et al. [67], who did not find any effect of furfural (0.18–1.43 g/L), 5-HMF (0.10–1.43 g/L), or acetic acid (9.0–11.6 g/L) in the enzymatic hydrolysis of 20% and 30% poplar wood chips previously subjected to thermal pretreatment.

Based on these previous studies, there is still no consensus on the extent of inhibition caused by these molecules on cellulases at high solids loadings, and therefore, further studies done under comparative conditions are necessary to identify which phenols, furan derivatives, and organic acids are inhibitory per se as well as their inhibitory concentration range. The involved mechanisms are also an open question.

#### Effect of lignin and pseudo-lignin on enzyme efficiency

In addition to these aforementioned inhibitors, lignin, which is a cross-linked phenolic and relatively hydrophobic macromolecule, has been considered detrimental to the action of cellulases. Studies carried out at low solids indicate that lignin would be a steric barrier to the enzymes [85–88] and/or would promote unproductive adsorption of cellulases through charged and noncharged interactions [30, 31, 76, 85, 87, 89, 90]. Moreover, depending on the type of biomass pretreatment, the structure of lignin can be changed and differently distributed in the remaining solid biomass, leading to different impacts on enzyme performance [27, 86]. Recently, studies on the inhibitory effect of lignin pretreated with different conditions have intensified due to controversial results of its effect on the hydrolysis of lignin-rich materials. Hao et al. [31] reported the adsorption/desorption of cellulases on lignin-rich residues obtained by dilute acid and dilute alkaline pretreatments of corn stover and its impact on the enzymatic hydrolysis of 2% pretreated material. These authors found that both pretreated biomasses exhibited inhibitory effects toward the cellulase preparation Celluclast^®^ 1.5 L plus Novozyme^®^ 188, with the highest inhibitory effect arising in the acid-pretreated biomass, which was probably due to its higher lignin surface content and stronger adsorption capacity. In agreement with these works, Kim et al. [57] reported the unproductive adsorption of cellulases on lignin as one of the limiting factors in high solids loadings.

However, there is no agreement regarding the detrimental effect of lignin. For instance, no effect was observed in the enzymatic hydrolysis of microcrystalline cellulose at 2% upon the addition of 10–35 mg of corn stover purified lignin per gram of cellulose [68] or of AFEX pretreated corn stover at increasing amounts of solids of 13.8, 19.3, and 24.9% [91]. Moreover, results from the enzymatic hydrolysis of 1% sugarcane bagasse treated by ball milling, a physical pretreatment that decreases cellulose crystallinity while keeping the native biomass composition, showed hydrolysis yields as high as 91% following 24 h of hydrolysis [92]; other researchers have also reported high biomass conversion yields in the presence of lignin [77, 93, 94]. Huang et al. [94] observed that the hydrolysis yield was improved by 8–12% or decreased by 6–16% depending on the biomass source and pretreatment conditions that produced lignins with different characteristics. These authors characterized each biomass and suggested that the lignin inhibition or stimulation effect is controlled by lignin hydrophobicity and the negative zeta potential, respectively. Still, Coffman et al. [93] reported that the enzymatic hydrolysis of Avicel was faster in the presence of lignin. It is important to emphasize that these reports used an enzyme preparation containing LPMOs, which could be responsible for the boosting effect, as suggested by Cannella et al. [77]. These authors evaluated the effect of lignin on LPMO activity in hydrolysis experiments using 30% hydrothermally pretreated wheat straw (a lignin-rich material) and concluded that lignin was able to activate LPMOs by behaving as a reducing agent, indicating a link between the oxidative breakdown of cellulose and redox cycles in lignin.

Therefore, the role of lignin in the enzymatic hydrolysis of a biomass can be detrimental, helpful, or neutral. These different effects are subjected to the biomass type that is associated to the natural lignin heterogeneity as well as to the pretreatment type and conditions undergone by the lignocellulosic material affecting lignin hydrophobicity and distribution in the pretreated material.

In addition, a lignin-like structure called pseudo-lignin [95, 96] and/or humins [97, 98] has been described to negatively affect the enzymatic conversion of pretreated biomass at low-solids content [96, 99, 100]. These aromatic polymers are formed by the polymerization of carbohydrate degradation products derived from acidic and heat-based pretreatments of lignocellulosic biomass, mainly conducted at severe conditions [95–97, 99]. Pseudo-lignins were described to negatively affect the enzymatic conversion of pretreated biomass through the formation of a physical barrier hindering the access to cellulose and/or by the unproductive binding of enzymes to its structure [98–100]. Hu et al. [99] showed that pseudo-lignin mixed to poplar holocellulose resulted in a strong inhibition of 9.5% to 25.1% on the overall enzymatic hydrolysis of cellulose, while Kumar et al. [100] showed that even small amounts of pseudo-lignin (5 wt% of cellulose) resulted in a significative decrease of 19.6% in cellulose conversion after 72 h reaction. Recently, He et al. [101] showed that pseudo-lignin reduced the activity of cellulolytic enzymes through non-productive adsorption due to the hydrophobic nature of the polymer. However, the nature of the pseudo-lignin and the exact mechanism for its inhibition effect on cellulases is not completely understood. It is suggested that pseudo-lignin surface charge and hydrophobicity may impact on its inhibitory effect [98]. In addition, the effect of pseudo-lignin in high-solids conditions still lacks investigation.

Thus, the enzymatic cellulolytic system may be affected by different inhibitors, decreasing the initial hydrolysis rate and the final sugar yield. Even though the inhibitory effect of glucose accumulation on cellulase activity has been widely accepted, more studies correlating the inhibition effect with the impact of other parameters, such as water availability for adequate diffusion and the rheological properties of the slurry, are necessary at high solids loadings to better understand their impact and contribution to the high-solids effects. Moreover, more attention should be given to the effect of oxidized products derived from the action of LPMOs, as these enzymes are present in novel enzymatic formulations, and to the effect of pretreatment-derived molecules, as the number of studies that have increased the solids loading on the pretreatment step has been rising in recent years.

### Mass transfer limitations in high-solids lignocellulosic slurries

Lignocellulosic slurries are classified as non-Newtonian fluids with a shear-thinning or pseudoplastic behavior (i.e., the viscosity decreases with an increase in the shear rate). This characteristic is attributed to the organization of fibers in the flow direction under higher shear rates [102]. Moreover, the apparent viscosity of these mixtures significantly increases with an increase in solids loading [103]. This behavior can be attributed to the tendency of fibers to build interjunctions when in suspension; therefore, when working with a high content of insoluble solids, the number of junctions would increase per area, and a higher amount of force would be necessary to break up the junctions for the flow to begin [102]. In addition, as the amount of free water is very limited, the lubricity between fibers decreases as the friction increases, adding more difficulty to the flow of the medium [24].

The observed increase of viscosity at high solids loadings results in a limitation in the mass transfer within the reactor [104]. This effect can have severe consequences on the process, as it results in poor contact between enzymes and substrate, which is necessary for adequate hydrolysis, and in the local accumulation of sugars near the enzymes, which increases the previously discussed feedback inhibition effect. The effect of viscosity has a more significant impact in the first moments of hydrolysis, when the fibers are still mostly intact; as the hydrolysis progresses, the enzyme action results in the liquefaction of the slurry, with a reduction in both water-insoluble solids [56] and in fiber length [105], yielding a reduced apparent viscosity and better flow properties.

It has been questioned whether the mass transfer limitation or the inhibitory effect of sugar accumulation was the main factor responsible for the reduction in hydrolysis yields with the increase of solids loading [24, 104]. Kristensen and coworkers [24] investigated the hydrolysis of filter paper at 20% (w/w) and at 5% (w/w) with the addition of an initial glucose concentration to match the final concentration reached in the 20% loading process. Their results indicated that a high glucose concentration was sufficient to account for the observed yield reduction when increasing the solids loading from 5 to 20%. However, when focusing on the first hours of hydrolysis, when the viscosity of the slurry is higher and the mass transfer limitation is greater, Du and collaborators [104] observed that higher solids loadings produced lower conversion yields even when releasing very little glucose, indicating that mass transfer was indeed the limiting factor in the studied conditions. This hypothesis was strengthened by the increase in hydrolysis yields with increased mixing speed for high solids loadings. Mass transfer limitations have been shown to play an important role in the reduced yields found for the hydrolysis of lignocellulosic biomass at high solids loading since the increase in mixing speeds resulted in higher mass transfer coefficients and higher hydrolysis yields [106].

Because the increased viscosity in high solids loadings can have a severe impact on the hydrolysis process, the rheological characterization of slurries can be a valuable technique for selecting better process parameters [93, 102, 107, 108]. However, this characterization is not easily carried out; representative sampling can be a challenge along with the loading of the rheometer due to the heterogeneous nature of the lignocellulosic suspension, with different fiber lengths and particle sizes [102, 107]. The choice of rheometer geometry also has an impact on the obtained data, as fibrous slurries are subject to wall slip and fracture, among other problems, which hinder the accurate measurement of rheological parameters [109]. In this context, the vane and the roughened parallel plates have been found to be the most adequate geometries for studying lignocellulosic materials [102, 107]. More recently, the online measurement of rheological properties has been suggested with impellers connected to a torque meter, resulting in more representative measurements as no sampling is needed [110]. Only the viscosity and yield stress can be determined in these systems, but, because these are the main parameters currently determined for lignocellulosic suspensions, this should not be seen as a limitation of the online technique.

Several factors that are closely linked to the biomass source and pretreatment type have been reported as having an impact on the rheological behavior of slurries, such as the water-retention capacity, the length and chemical composition of fibers, and the inter-fiber interactions [24, 111, 112]. Specifically, the impact of the length of the fibers in the viscosity and yield stress of the lignocellulosic slurry is controversial. For instance, corn stover subjected to three pretreatments was investigated in a study that compared the yield stress and viscosity of the different samples during enzymatic hydrolysis. Deacetylation with mechanical refining pretreatment provided the highest yield stress reduction and the highest glucose production of 157 g/L when working at a 32% solids loading, even though it had a higher mean fiber size when compared to the dilute acid pretreatment [108]. This observation is in agreement with the data of Wiman et al. [102], who reported better results using biomass samples with bigger particle sizes, while a reduction in particle size had a negative effect on the increase in viscosity, possibly due to an increase of junction points per area and a narrower particle size distribution. However, other studies have reported a decrease in viscosity and yield stress when the particle size was smaller as an effect of loss and decrease of fiber macropores, leading to a lower water constraining capability and, therefore, to more free water in the medium [113, 114]. The severity of pretreatment also affects the rheological behavior of fibers; with increasing severity, the composition of the biomass changes, and a decrease in particle size, is also observed [113, 114]. These confounding effects can lead to mistaken conclusions about the impact of particle size on the viscosity and yield stress of biomass slurries.

The chemical composition of the chosen biomass may also have a significant impact on the rheological characteristics of the hydrolysis mixture and, therefore, may affect the maximum solids loading that can be used. For instance, different torque and power consumption values were observed when mixing dilute acid-pretreated *Arundo donax* and spruce slurries, and surprisingly, the mixing energy input was independent of the initial water-insoluble solids content for *Arundo donax*, while it became higher with increasing amounts of spruce. This discrepancy was attributed to the different amounts of lignin present in the lignocellulosic materials, of 36.8% in *Arundo donax* and of 46% in spruce [115]. As hemicelluloses have a high water-constraining capacity [116], their presence in the lignocellulosic biomass also influences the rheological properties of the material. Slurries formed by lignocellulosic materials with a higher hemicellulosic content usually show increased viscosities, as observed by Ludwig and collaborators [117] when comparing pretreated beech wood and wheat straw. The study described that the pretreated wheat straw had a higher hemicellulosic content (26%) compared to pretreated beech wood (6.8%) and, therefore, showed a swelling behavior that increased the viscosity of the hydrolysis medium.

The rheological behavior of hydrolysis slurries is thus an important characteristic to be assessed for the processing of lignocellulosic biomass at high solids loadings, as it can be used to develop processes, design reactors and impellers, and assess the energy required for stirring. Moreover, it can guide the choice of biomass and pretreatment to produce slurries with more adequate rheological characteristics. However, the accurate determination of rheological properties for such a heterogeneous material remains a challenge, and future studies should focus on developing more adequate equipment to measure these parameters.

## Advances in high-solids enzymatic hydrolysis

Several of the limiting factors that were addressed in the previous section (i.e., the water constraint effect, the decrease in enzyme effectiveness by inhibition or adsorption, and the difficulties in mixing and mass transfer due to the rheological characteristics of the reaction media) have been targets of studies seeking to develop strategies to overcome those limitations and, by extension, to take greater advantage of operating at high solids loadings. Many aspects can be optimized to improve the efficiency of the enzymatic hydrolysis at high-solids conditions; the more noteworthy are the enzyme formulation, the biomass feeding strategy in the reactors, the supplementation of the media with additives, the design of reactors, and the strategies for separation and detoxification of streams [77, 117–120]. This section will address advances in enzyme activity and formulation to overcome inhibition, improve liquefaction, and increase the final hydrolysis yields; advances in biomass fed-batch strategies to overcome the overload of solids at the onset of the reaction and, therefore, improve the rheological properties of the reaction media; and advances in the design of reactors and impellers that tackle the challenges of mixing and heat and mass transfer limitations in high-solids conditions.

In addition, one important issue, seldom addressed, regards the type of pretreatments that would be better suited for the operation at high solids loadings. The enzymatic hydrolysis studies that have evaluated the effectiveness of different pretreatments for a given type of biomass are usually carried out at low-solids conditions [8, 121–125], as it has been frequently assumed that pretreatment methods would have comparable efficacies independently of the solids content in the hydrolysis media. However, studies at low solids do not allow the direct use of the conditions optimized in a high-solids reaction medium, which has different physicochemical properties that affect the interactions of biomass with water and enzymes.

Indeed, a recent study by Weiss et al. [48] showed that the hydrolysis yields at low solids were not indicative of the performance of a given pretreatment at a higher content of solids, particularly after reaching the point when free water disappeared from the system. The authors compared the enzymatic hydrolysis of wheat straw pretreated with a combination of steam pretreatment followed by either delignification or treatment with xylanases. The results showed that the delignified sample showed a better digestibility at low solids, while the sample treated with xylanases resulted in better yields at high solids; these results were correlated to the different ability of the pretreated biomass to constrain water at high-solids conditions. As pointed out by the authors, these findings highlighted the importance of comparing the efficiency of different pretreatments at high solids for the acquisition of industrially relevant data that would allow a better choice of the pretreatment type and working conditions. Therefore, one strategy to reduce the high-solids effect would be to identify the most suitable pretreatment for the target biomass with the aim to improve its physicochemical properties in a high-solids environment. However, there is a lack of reports on how changes made to the lignocellulosic materials by different pretreatments affect the interactions of the biomass with water and enzymes at high solids loadings. This knowledge gap hinders the discussion on this topic, and therefore, it will not be addressed in this section.

### Improvements in the formulation of enzymes

Several strategies to improve the efficiency of enzymes and to diminish inhibition effects at high-solids conditions have been reported. These investigations focused on the use of adequate enzyme loadings and new enzyme formulations with complementary activities (laccases, hemicellulases, pectinases, and LPMOs) in addition to the engineering of proteins to achieve resistance to inhibitors [24, 37, 56, 67, 75, 77, 126–128]. The improvement of the hydrolysis reaction upon the addition of non-catalytic proteins has also been studied [35, 129, 130].

The effect of increasing the enzyme loading as a strategy to alleviate the high-solids effect has been assessed in a relevant number of reports. One study evaluated the effect of the enzyme loading of the cellulase Spenzyme^®^ CP for the enzymatic hydrolysis of 15% (w/w) acid-pretreated corn stover using a response surface methodology [26]. The authors confirmed that an increase in the enzyme loading from 5.8 to 19.2 FPU/g cellulose improved the cellulose conversion and glucose concentration from 50% and 80 g/L to 70% and 110 g/L, respectively. In contrast, Kristensen et al. [24] concluded that it was not possible to overcome the high-solids effect and improve the hydrolysis yields when increasing the enzyme loading of a blend of Celluclast^®^ and Novozyme^®^ 188 from 5 to 20 FPU/g of substrate. Interestingly, another study pointed out that the use of high enzyme loadings could be detrimental to the process, as it would lead to competitive inhibition when substrate saturation is reached; consequently, part of the enzymes could be temporally non-productively adsorbed to the substrate [55].

Indeed, more important than increasing the enzyme dosage, the study of novel enzyme formulations was suggested to have a greater impact on alleviating the high-solids effect. It has been reported that an optimized enzyme blend with accessory enzymes (hemicellulases, pectinases, β-glucosidase, and laccases) and additives resulted in a low requirement of cellulase dosage, of only 4 FPU/g, to effectively hydrolyze 22% (w/v) alkali-pretreated sugarcane bagasse, achieving a glucose titer and yield of 122 g/L and 80%, respectively [127]. Ramos et al. [126] studied the impact of enzyme loadings with Cellic^®^ CTec 2 in the range of 1.14–18.16 FPU on the hydrolysis of steam-exploded sugarcane bagasse in high-solids conditions (20% w/w) and observed that conditions with intermediary loadings of 4.54 FPU/g of substrate could be used to achieve 76.8 g/L glucose, corresponding to a 69.2% yield. Thus, increasing the enzyme loading does not necessarily translate into linear gains in glucose concentrations and yields, as the performance of the enzymatic preparation in high solids loadings seems to be much more dependent on the composition of the formulation than on simply increasing the amount of enzymes in the reaction media.

Based upon the reports of studies that evaluated different enzyme formulations at high solids loadings [56, 67, 75], β-glucosidase (to avoid cellobiose accumulation), xylanases and β-xylosidases (to avoid xylooligosaccharide accumulation), and LPMOs (to increase the hydrolysis rates) were identified as acting in synergism with cellulases for an efficient conversion of lignocellulose polysaccharides. The requirement of high levels of β-glucosidase and xylanases was corroborated by Di Risio et al. [67], who evaluated the hydrolysis of thermally pretreated poplar wood chips at 20% and 30% of solids using Cellic^®^ CTec and Novozyme^®^ 188 combined with AlternaFuel AF100L and A200L, Accellerase 1500, and Multifect xylanase. These authors reported that the combination of 10% Cellic^®^ CTec, 1.5% Novozyme^®^ 188, and 5% Accellerase 1500 resulted in the highest glucose and xylose concentrations and the lowest accumulation of cellobiose when compared with several enzyme combinations.

Silva et al. [56] evaluated the hydrolysis of hydrothermally treated sugarcane bagasse at 5%, 15%, and 20% using the commercial enzymes Celluclast^®^1.5L plus Novozyme^®^ 188, Cellic^®^ CTec2 alone or blended with Cellic^®^ HTec, and a laboratory-made blend of enzymes from *Trichoderma reesei* Rut C-30 and *Aspergillus awamori*. These authors observed similar glucose yields in the range of 80–86% for all enzymes at 5% of solids, while Cellic^®^ CTec2 alone or blended with Cellic^®^ HTec had a better performance at 15% and 20% of solids and achieved higher yields of 72% and 69%, respectively, than Celluclast^®^ plus Novozyme^®^ 188 and the laboratory-made blend, which reached 45% and 57%, respectively, at 20% of solids. The study also evaluated the tolerance of these enzyme blends to glucose inhibition by adding exogenous glucose (30 and 60 g/L) at the beginning of the hydrolysis with 5% of solids. It was observed that, although the highest glucose concentration tested greatly affected the hydrolysis rates for all enzymes and resulted in the reduction of yields (from 42 to 60% depending on the enzyme), Cellic^®^ CTec2 showed higher tolerance to inhibition, particularly after 24 h of hydrolysis. Interestingly, although the laboratory-made formulation was less tolerant to the glucose background, it improved the rheology of the system, resulting in faster biomass liquefaction and, consequently, higher hydrolysis rates and yields within 6 h of hydrolysis for all solids loadings tested. Thus, it was concluded that the laboratory-made blend of *T. reesei* Rut C-30 and *A. awamori* could contain endoglucanases with a higher tolerance to sugar inhibition and/or a higher penetration and action on internal cellulose fibers, and/or contain accessory enzymes acting synergistically for better biomass liquefaction. In this sense, it is crucial to emphasize the importance of prospecting and identifying the enzymes involved in this faster liquefaction effect to improve the performance of currently used enzymes for high-solids hydrolysis.

In fact, one of the main concerns regarding enzymatic hydrolysis at high solids loadings is to quickly achieve biomass liquefaction to improve the availability of water and to reduce mass transfer limitations, which consequently improves the hydrolysis kinetics [93]. Endoglucanase activity has a special role in the biomass liquefaction, as many studies have reported the correlation of the decrease in viscosity with the use of high levels of endoglucanases [93, 131, 132]. In addition, other works have demonstrated the swelling effect of endoglucanases on celluloses [36] and on lignocellulosic materials [133]. Josefsson et al. [36] reported that a purified endoglucanase (Cel 17B) from *T. reesei* caused softening and swelling of a cellulose film, which was probably due to the reduction of restraining forces within the cellulose, while Pääkkö et al. [133] concluded that the addition of endoglucanases to softwood cellulose pulp promoted cell wall delamination and improved the enzymatic production of microfibrillated cellulose. Thus, the inhibition of endoglucanases consequently results in reduced biomass liquefaction, which impacts the sugar yields and imposes a significant limitation on the biomass conversion at high solids loadings.

Another strategy that could be explored is related to the improvement of endoglucanases by protein engineering. Reyes-Ortis et al. [37] constructed chimeric endoglucanases by fusing a carbohydrate-binding module with two thermophilic endoglucanases. They showed that the chimeric enzymes enhanced the enzymatic hydrolysis of 1% microcrystalline cellulose up to threefold. These authors suggested that the chimeric enzymes were able to penetrate and hydrolyze the bulk of the substrate due to the action of the carbohydrate-binding module, which created sites for water coupling and enzymatic access, leading to substrate swelling. However, in an environment without free water, it is difficult to predict the penetration capacity of these modified enzymes, so an evaluation of the benefits at high solids loadings using lignocellulose substrates is necessary.

Beyond the classic cellulosic biomass-degrading enzymes, some non-hydrolytic proteins, such as a fungi expansin-like protein called swollenin, have been shown to disrupt and loosen inaccessible regions of the well-organized crystalline cellulose structure by the non-hydrolytic weakening of hydrogen bonding in a process called amorphogenesis, thereby improving the accessibility of cellulases to cellulose [35, 129]. For example, the addition of a purified swollenin of *T. harzianum* to the blend of Celluclast^®^ and Novozyme^®^ 188 resulted in a twofold increase in the hydrolysis efficiency of hot water-pretreated Miscanthus biomass [129]. Another study showed that swollenin had a pronounced synergism with xylanases on the hydrolysis of steam-pretreated corn stover [134]. In contrast, Eibinger et al. [130] reported that a swollenin from *T. reesei* (SWO1) was essentially inactive on pure celluloses and had only a slight synergy with cellulases on untreated and mildly treated lignocellulose materials, while demonstrated a strong adsorption in xylan but with no synergism with xylanases. Interestingly, these authors observed a remnant hydration shell that surrounded the cellulose nanocrystals incubated with SWO1 after drying the material. This suggests that the maintenance of a hydration layer by a protein could be of interest for hydrolysis at high solids loadings, as these proteins could cover hydrophobic spots, which are exposed in an environment without free water, and mitigate the unproductive binding of cellulases. However, there is a lack of studies reporting the use of enzyme cocktails containing high levels of swollenin for the high-solids hydrolysis of lignocellulose materials. Thus, although the supplementation of enzyme formulations with swollenin and expansin-like proteins seems promising, studies to assess the benefits of those proteins at high-solids conditions are still needed.

Moreover, the addition of other enzymes has been recommended to diminish the inhibitory effect of oligomeric sugars, phenolics, and lignin. In this sense, Xue et al. [62] reported that enzyme blends with Multifect^®^ pectinase showed the highest conversion of recalcitrant oligosaccharides, which were described to have a stronger inhibitory effect on cellulases. Tejirian and Xu [83] reported that the addition of tannases from *Aspergillus oryzae* (25 mg/L) in a cellulase blend could mitigate the inhibition effect of tannic acid (1 mM) on the hydrolysis of 4.5% dilute acid-pretreated corn stover. These authors suggested that the added tannases hydrolyzed the tannic acid and, consequently, deprived the oligomeric phenolic’s ability to complex with and/or inactivate cellulases. In addition, hydrolytic enzymes and non-hydrolytic enzymes, such as laccases, lignin-peroxidases, and manganese peroxidases, have great potential to improve enzyme blends. However, there are controversial reports of enzymatic hydrolysis using these oxidative enzymes to supplement cellulase blends, as positive and negative effects on yields were observed depending on the lignocellulosic substrate used [128, 135]. As an example, Raj and Krishnan [128] reported an increase in glucose concentration from 119 to 157 g/L for the hydrolysis of ammonia-pretreated sugarcane bagasse when supplementing Cellic^®^ CTec2 with exogenous laccase (200 U/g solids) and 1-hydro-xybenzotriazole (HBT) (25 mg/g solids) as a mediator. In contrast, the presence of laccase in the enzymatic hydrolysis of Sigmacell with exogenous lignin reduced the glucose yield by 37% when compared with control assays without laccase [135]. These authors suggested that the oxidation of lignin could lead to the formation of phenolic oligomers, which affect endoglucanase, cellobiohydrolase, and xylanase activities, as discussed in the inhibition section. Thus, the supplementation of cellulolytic blends with these oxidative enzymes should be evaluated for each material.

Currently, LPMOs are of great importance as these enzymes are already supplemented in commercial blends. These oxidative enzymes have been described as able to boost the lignocellulose conversion, especially at high solids loadings. For instance, the higher glucose yields reported by Silva et al. [56] using Cellic^®^ CTec2 were discussed to be related to the presence of significant levels of LPMOs, as similarly reported by Cannella et al. [77], who showed a production of up to 4% of oxidized glucose during the hydrolysis of 30% hydrothermally treated wheat straw with Cellic^®^ CTec2. Hu et al. [75] replaced part of the cellulase loading by xylanases and LPMOs for the hydrolysis of steam-pretreated poplar and corn stover at 2%, 10%, and 20% of solids, demonstrating that high xylanase loadings and low amounts of LPMOs enhanced the hydrolytic performance of cellulases during the hydrolysis of corn stover at 20% (w/v). In addition, when the authors compared an optimized formulation containing Celluclast^®^ 1.5 L, xylanase, and LPMOs to the Cellic^®^ CTec 2, which contains LPMOs, similar hydrolysis yields were obtained (70% and 75%, respectively). However, the use of Cellic^®^ CTec3, an improved cocktail in comparison to Cellic^®^ CTec 2, resulted in a 90% hydrolysis yield [75], which confirmed that the adequate formulation of enzyme preparations with accessory enzymes could accelerate the hydrolysis rate and improve yields, being a strategy of decisive importance for hydrolysis at high solids loadings.

Nevertheless, it is important to point out that one of the concerns about LPMO’s supplementation is related to the oxygen dependence of the reaction process, as the removal of oxygen leads to a strong inhibition of LPMOs [78, 136]. This oxygen dependence not only adds on the complexity of applying LPMOs but also impacts the choice of process type for the production of cellulosic ethanol. For instance, lower yields of ethanol production were observed when comparing simultaneous saccharification and fermentation (SSF) with separate hydrolysis and fermentation (SHF) in the hydrolysis of wheat straw at 30% solids loading with Cellic^®^ CTec2, which was probably due to the competition between LPMOs and yeasts for dissolved oxygen in SSF [78]. The authors also evaluated different loadings of Cellic^®^ CTec2 of 15 or 22.8 mg/g of cellulose and compared its performance with the Celluclast^®^ plus Novozyme^®^ 188 blend in both process strategies. Interestingly, although the presence of LPMOs boosted the hydrolysis and the entire process, excessive amounts of Cellic^®^ CTec2 led to the formation of significative concentrations of gluconic acid, which cannot be fermented by the yeast and result in a loss of ethanol formation.

In summary, the high-solids enzymatic hydrolysis has taken advantage of recent findings related to the development of enzyme molecules and enzyme formulations with increased efficiency for the hydrolysis of cellulose. These enzyme formulations also have accessory activities to curb the accumulation of inhibitors such as cellobiose and hemicellulose-derived molecules. However, in spite of the progress that has been made by seeking the most effective enzyme blend, this core theme is still not fully covered. In fact, studies on less-expensive tailor-made blends with increased efficiency for a high-solids milieu are desirable, considering the diversity of lignocellulosic biomass coupled to the different modifications promoted by pretreatment methods.

### Use of additives for hydrolysis improvement

In addition to the aforementioned advancements in enzyme formulations to cope with the high-solids effect, several additives, such as Tween 20 [127, 137–139], Tween 80 [140], bovine serum albumin [119, 138, 141], lignosulfonate [142], and polyethylene glycol (PEG) [57, 78, 119], have been described to have beneficial effects upon enzymatic hydrolysis rates and yields. Their mechanism of action in enzymatic hydrolysis is not completely understood. Bhagia et al. [138] cited a total of ten different mechanisms that were proposed to explain how additives could increase enzymatic hydrolysis of cellulose. However, these additives are mainly thought to act through two mechanisms: (i) preventing cellulase deactivation at air–liquid interface during the hydrolysis by forming a network at the surface and reducing the surface available for enzymes [138, 139, 143] and/or (ii) lowering the non-productive adsorption of cellulases to lignin, increasing the cellulases availability and reducing the enzyme-loading requirements to achieve relevant sugar yields [137, 140].

Bhagia et al. [138] showed that cellulases deactivation at the air–liquid interface could be the main cause of cellulose conversion decrease during hydrolysis conducted using shaking flasks with low-enzyme loading. These authors evidenced a significant increase in enzymatic hydrolysis of different samples of pretreated poplar (1% w/v of glucan) when the surfactant Tween 20 was added, independent of the lignin content. Thus, the authors labeled this additive as “surface-active additive” as it had a higher surface activity than cellulases and proved to reduce the interfacial deactivation of enzymes. In another study, Bhagia et al. [139] showed that Avicel conversion at high solids loading (15% w/v of glucan) more than doubled when adding Tween 20 to a reaction medium in shaking flasks with low enzyme dosage (approximately 2.5 FPU/g of glucan using Acellerase^®^ 1500).

Although it has been proposed that surfactants would be responsible for the preservation of enzyme activity and stability, most of the studies of improvement by surfactants correlate it to the prevention of the unproductive binding of enzymes to lignin. One study showed that the addition of 5 g/L Tween 20 significantly improved the rates and yields of the hydrolysis of 5% pretreated wheat straw, particularly for samples with a high lignin content [137]. Indeed, the positive effect of Tween 20 was more prominent for the hydrolysis of dilute acid-treated wheat straw, resulting in an increase of up to 23.2% in cellulose conversion, while its supplementation to the hydrolysis of samples pretreated by oxidative methods, which mainly aim at delignification, resulted in no effects or only discreet improvements (− 0.9 to 6.2%). Thus, the authors suggested that the benefits of adding Tween 20 were dependent on the pretreated biomass composition, as this additive aims at changing lignin hydrophobicity and surface charges, helping to alleviate the adsorption of cellulases. The interaction effect of Tween 80 with Cellic^®^ CTec 2, Cellic^®^ HTec 2, and laccase in the hydrolysis of 8% washed and unwashed ammonia-pretreated sugarcane bagasse was evaluated using a central composite design [140]. The authors reported optimal loadings for the enzymes and Tween 80 per mass of glucan, achieving hydrolysis yields of 84.30% and 97.10% for washed and unwashed substrates, respectively. The highest increase of 75.85% on cellulose conversion was observed for the unwashed substrate, when compared to 12.74% for washed substrates, suggesting that tailored-made enzyme blends applied with additives could substitute the need for the removal of inhibitory compounds. Therefore, the benefits of the addition of Tween 80 to the study conducted by Oladi and Aita [140] could be related to the circumvention of the interactions of enzyme with lignin, thus making available a higher amount of enzyme in a medium containing a high concentration of inhibitory compounds.

In another extensive study, Xu et al. [127] evaluated the loadings and interactions of enzyme and additives (Cellic^®^ CTec3, β-glucosidases, laccases, pectinases, hemicellulases, and noncatalytic proteins and several nonionic and ionic surfactants) for the hydrolysis of 10% alkali-pretreated sugarcane bagasse, followed by a fed-batch hydrolysis strategy to reach 22% of solids (w/v). The optimized blend of Cellic^®^ CTec3 with other enzymes (150 U hemicellulases and 60 mg β-glucosidases per gram of dry matter) and additives (25 mg non-catalytic whey protein powder, 25 mg of biosurfactant sophorolipid, 40 mg Tween 80, 10 mg ionic surfactant calcium lignosulphonate per gram of dry matter) allowed the reduction of the cellulase dosage to 4 FPU/g for the hydrolysis of 20% of solids (w/v) with hydrolysis yields and glucose concentration of 80% and 122 g/L, respectively. In accordance with those data, Cannella and Jørgensen [78] reported that PEG3000 (0.01 g/g of dry matter) could be used to decrease the enzyme loading by up to 30% for the saccharification of 30% hydrothermally pretreated wheat straw at different process conditions (SHF, SSF, or pre-saccharification followed by SSF) with no detrimental effect on the efficiency of the process.

New enzyme formulations added alongside additives that prevent cellulase deactivation at the air–liquid interface of reactions or lower the non-productive adsorption of cellulases to lignin have the potential to not only increase the conversions but also impact the overall amount of enzyme required for effective hydrolysis, leading to a reduction in costs.

### Fed-batch strategies

Fed-batch strategies have been reported to overcome the mixing difficulties and the decreased yields of the high-solids enzymatic hydrolysis due to the maintenance of a low viscosity in the hydrolysis system, with obvious advantages regarding the mass transfer of substrate and enzymes, and the mixing power input requirement in comparison to the one-step batch mode [118, 127, 144–148]. Studies regarding the setup of the fed-batch strategy have evaluated the initial solids loading, the number and periodicity of substrate feeding, and the batch or fed-batch enzymes addition. Table 1 presents the differences in the working conditions (biomass and pretreatment type, enzyme dosage, among others) in fed-batch studies.

**Table 1:** Parameters that were evaluated and the resulting hydrolysis yields for fed-batch operations at high-solids loading

Biomass	Pretreatment	Enzyme	Enzyme dosage	Feeding strategy	Time of feeding (h)	Final solids loading (%)	Enzyme addition mode	Time (h)	Glucose yield (%)	References
Sugarcane bagasse	Low-temperature aqueous ammonia soaked	Cellic^®^ CTec2	10 FPU/g DM	12% + 8% + 5% + 5% + 5% + 5%	3, 8, 12, 18, 24	40	Whole	96	~ 62	[128]
Sugarcane bagasse	Alkali	Cellic^®^ CTec3	4 FPU/g DM	10% + 5% + 4% + 3%	8, 12, 16	22	Whole	72	76	[127]
Sugarcane bagasse	Alkali organosolv	Cellic^®^ CTec2	3 FPU/g substrate	8% + 4% + 4% + 4%	6, 12, 18	20	Whole	72	51	[118]
Sugarcane bagasse	Alkali	Cellic^®^ CTec2	10 FPU/g substrate	12% + 7% + 7% + 7%	6, 12, 24	33	Whole	120	~ 60	[144]
Sugarcane bagasse	Formiline	Cellulase from Novozymees	10 FPU/g DM	6.66% + 6.66% + 6.66%	12, 36	20	Whole	210	80	[146]
Corn stover	Steam explosion	Cellic^®^ CTec2	10 FPU/g glucan	15% + 7.5% + 7.5%	12, 24	30	Whole	120	60	[156]
Corn stover	Dilute acid	Cellic^®^ CTec2 + Cellic^®^ HTec2	5 FPU/g substrate	9% + 3% + 3%	12, 24	15	Whole	72	76	[145]
Corn stover	Steam explosion and NaOH–H_2_O_2_	Cellulase from Sunson	20 FPU/g TS	12% + 6% + 6% + 6%	12, 36, 60	30	Whole	144	~ 58	[72]
Corn stover	Dilute acid	Spezyme CP	10.7 FPU/g cellulose	Maintaining 15% of solids	Every 24	25	Split	288	80	[159]
Rice straw	Dilute acid	SacchariSEB-C6	3 FPU/g TS	10% + 5% + 5%	4, 8	20	Split	48	~ 77	[147]
Rice straw	Dilute acid	Cellic^®^ CTec2	15 FPU/g glucan	10% + 10% + 10%	12, 24	30	Whole	60	76	[151]
Sugarcane straw	Hydrothermal	Cellic^®^ CTec2	10 FPU/g substrate	2.5% + 2.5% + 2.5% + 2.5% + 2.5% + 2.5% + 2.5% + 2.5% + 2.5% + 2.5% + 2.5% + 2.5%	1, 2, 4, 8, 12, 18, 24, 30, 36, 42, 48	30	Split	72	~ 60	[148]
Corncob	Dilute acid with alkali	Cellulase from Sino Biotechnology Co.	10 FPU/g DM	5% + 5% + 5% + 5%	6, 12, 18	20	Split	72	59	[104]
Brewers spent grains	Dilute acid hydrothermal	Cellic^®^ CTec2	10 FPU/g biomass	12.5% + 12.5%	24	25	Whole	48	~ 35	[155]
Barley straw	Steam	Celluclast^®^ + Novozyme^®^ 188	7.5 FPU/g DM	10% + 15%	24	15	Whole	72	68	[154]

The onset of the fed-batch process should use the highest initial solids loading that the system can hold to promote fast liquefaction with a continuous glucose release before beginning the biomass feeding strategy. Raj and coauthors [128] tested the effect of the initial solids loading of aqueous ammonia-pretreated sugarcane bagasse in the range of 6–16% and observed that the use of 14% solids and 10 FPU/g of dry material provided the highest accumulation of glucose coupled to a visible liquefaction effect. These results were similar to the data reported by a study that compared the initial solids from 9 to 18% of alkali-pretreated sugarcane bagasse and 8.5 FPU/g of substrate and observed that the highest glucose concentration was obtained with 15% of solids [149]. However, when also studying alkali-pretreated sugarcane bagasse, Gao and coauthors [144] observed a decrease in the glucose concentration and yield when increasing the initial solids loading from 12 to 14%. Mukasekuru et al. [118] chose an optimal initial solids loading and enzyme dosage of 8% and 3 FPU/g of substrate, respectively, to enable rapid liquefaction and maximum glucose concentration in the first 6 h (42 g/L), while an extension in the liquefaction time to 12 h was observed when the solids loading was above 10%. This discrepancy in the optimal initial solids loadings reported by the several studies dealing with similar substrates may be due to the use of different enzyme loadings and different pretreatment and/or hydrolysis conditions, indicating that this parameter needs to be optimized in a case-by-case basis. Nevertheless, when comparing the different studies shown in Table 1, it is possible to conclude that an initial solids loading in the range of 8–15% would be a cautious setting to work, resulting in a low viscosity media and a fast liquefaction step.

Another important parameter to be studied to lessen the impact of high solids loadings is the number and periodicity of substrate feeding, as it impacts the glucose yields and the process viability on an industrial scale. As shown in Table 1, different numbers and periodicity of feeding events are approached by the authors. Raj et al. [128] evaluated the time of feeding ranging from 22 to 54 h and observed that the highest glucose and xylose concentrations were obtained for the 22 h feeding. An earlier feeding time of 18 h (51% yield) was also favorable in comparison to 30 h (45% yield) [118] and was in accordance with another study that reported 16 h as ideal [127]. It is likely that an earlier feeding time is favored due to the loss of enzymes activity observed in extended periods of enzymatic hydrolysis [118, 128].

Short substrate loading intervals were also evaluated. Thus, feeding periodicities of 0, 1, and 2 h were compared to 0, 4, and 8 h, showing that the longer intervals resulted in a better conversion of cellulose [147]. Nevertheless, the longer periodicity feeding of 8 h was quite small in comparison to the aforementioned time intervals. A similar result was found for a different approach that evaluated a short periodicity of 5- and 10-min feedings and small amounts of substrate. The 10-min feeding time resulted in higher glucose titers as, according to the authors, the 5-min feeding was too short to allow for biomass liquefaction [150]. In spite of the results that indicate that the number and periodicity of substrate feeding influence the fed-batch performance, it has also been reported that they are not significant for the final saccharification yields and glucose concentration [146, 151]. All in all, the feeding time interval depends on the required time for biomass liquefaction with the aim of keeping a low viscosity media and hydrolysis rate throughout the entire fed-batch process and will also be highly dependent of the hydrolysis conditions used.

The choice of enzyme feeding (i.e., the loading of the total enzyme at the onset of the enzymatic hydrolysis or the enzyme addition in fresh batches) is also a common parameter evaluated in fed-batch processes. The obvious advantage of the enzyme addition in batch mode is the liquefaction and a higher initial hydrolysis rate provided by the higher enzyme/substrate ratio. Indeed, the batch addition rapidly resolves the water constraint hurdle and anticipates the liquefaction effect when compared to the splitting addition of enzymes [74]. However, it must also be taken into account that the one-batch enzyme addition subjects the enzymes to activity loss over time, shear forces, air–liquid interface, and high temperatures, in addition to the unproductive binding to lignin [138, 139, 143, 152, 153].

Enzyme feeding was tested by adding 50%, 25%, and 25% in three batches of fresh enzymes in each feeding of the substrate, resulting in a discrete 1.9% improvement in glucan conversion only after 48 h of hydrolysis [147]. Du and coauthors [104] also observed a slight improvement, of 4%, in glucan conversion using the split addition of enzymes after 72 h of enzymatic hydrolysis; however, the improvement observed when shifting from the one-batch to the fed-batch addition of substrate was much more impactful, of 19%. Both studies reported that the improvement observed by the splitting of enzymes is dependent on time, i.e., during the initial phase of hydrolysis, the one-time feeding strategy of enzymes obviously and rapidly decreased the viscosity of the media, while the proportional addition of enzymes provided a better glucose release toward the end of the hydrolysis. However, the reported improvements are quite low and might not be enough to justify a more complex process over a one-time addition of enzymes.

Moreover, some studies have shown that the split addition of enzymes may even result in lower glucose yields when compared to the one-batch addition strategy [74, 145, 151]. In these assays, the low viscosity effect provided by the whole addition of the enzymes to the hydrolysis media likely compensated the loss of enzymatic activity. In addition, Cardona and coauthors [74] suggested that an inhibitory effect in enzymes is unavoidable, even when splitting enzyme additions. Finally, some works reported no difference in the final sugar yields when adding the cellulases all at once or using a split approach after 72 h of high-solids enzymatic hydrolysis using a fed-batch operation [128, 144, 146, 154]. In light of these results, the split addition of enzymes does not seem to be an effective way to significantly increase the hydrolysis yields in fed-batch processes.

When comparing fed-batch with the batch addition of substrate, it has been shown that, when working with 15% or less of total solids, the fed-batch has no impact or even a negative effect when compared to the batch operation [104, 153, 154]. However, for higher solids concentrations, the fed-batch strategy usually improves the hydrolysis yields. Some studies reported slight improvements when working with 20% of total solids, reaching glucose conversion improvements of 1% and 2.2% at 48 and 72 h of hydrolysis, respectively, in comparison to the batch mode [104, 155]. More prominent results were reported by Liu et al. [156] for the fed-batch using a periodic peristalsis reactor as it increased the hydrolysis yield by 13.9% with a 30% solids loading compared to that from the batch mode. Similarly, increases in cellulose conversion of 13% and 12% were respectively observed when working with 20% and 30% of solids loading in fed-batch results in comparison to the batch mode after 72 h of hydrolysis [148].

Fed-batch has also been shown to improve the yields obtained with an SSF configuration at high solids loadings when ethanol is the desired product [157]. It was observed a high cell mortality when the SSF process was conducted in batch mode, which was attributed to higher accumulation of inhibitors, higher osmotic stress and/or less-efficient mass transfer when compared to the fed-batch operation at a high solids loading of 22%. A similar effect was observed by López-Linares et al. [158], who reported low cell viability in SSF mode at high-solids conditions. These authors reported better results with the SHF configuration; however, they did not test the fed-batch approach. The choice of SSF over SHF when working with high solids loadings is still an open-matter, as the first not only affects cell viability, but may also cause a decrease in the action of LPMOs, as discussed in the previous section.

The fed-batch operation has been proven to be an efficient strategy to maintain a low viscosity in high-solids content; however, keeping the viscosity low will not directly translate into increased sugar titers. Nonetheless, the improvement in mass transfer obtained by the constant viscosity of the media could alleviate the power input required for mixing.

### Reactor and impeller design

The lignocellulosic biomass hydrolysis mixture with high-solids content has a high viscosity that causes mass transfer limitations and impairs the progress of the hydrolysis reaction. The improvement in the interaction between substrate and enzymes realized through better mixing would allow greater liquefaction of the fibrous matrix in the first hours of hydrolysis, reducing the mixture viscosity and resulting in better mass transfer in the system [160].

Better mixing can be obtained by simply increasing the mixing rate, a strategy that has been shown to improve hydrolysis yields when using both shaken flasks and bioreactors. Ramos and coworkers [126] found a positive correlation between glucose production and the agitation intensity of shaken flasks when studying the enzymatic hydrolysis of steam-exploded sugarcane bagasse at increasing solid loading up to 20%. When working with 20% (w/v) corn stover pretreated by steam explosion, Wojtusik and collaborators [106] observed that the increase in the impeller speed from 50 to 250 rpm resulted in higher hydrolysis yields. The authors correlated these results with the increase in the mass transfer coefficient at higher mixing rates. However, it has been shown that an increase in mixing forces in the reaction mixture can cause enzyme deactivation, hampering the enzymatic hydrolysis instead of helping it [152]. This was mainly attributed to turbulent normal stresses, which are forces that act perpendicular to the enzyme molecule, causing it to unfold [161]. The degree of deactivation in a given shear rate was found to be different depending on the impeller used, so this effect could be minimized with the right choice of stirrer [161]. Therefore, further study of this phenomenon could guide the design of reactor impellers that minimize the turbulent normal stresses while yielding adequate mixing at high-solids loadings. However, according to Lou and coworkers [162], there has been some indication that high-solids loadings stabilize the enzymes in high mixing forces.

Even at increased agitation intensities, shaken flasks have been deemed unsuited for tests at relevant industrial conditions of high-solids loading due to the poor mass transfer properties. Therefore, for lab-scale trials evaluating parameters such as enzymatic mixture and pre-treatment conditions, alternative systems have been proposed. Caspeta and collaborators [163] studied a mini peg mixer reactor that yielded 1.33 higher glucose titers than shaken flasks. However, this system may not be easily set up and would limit the amount of simultaneous testing being conducted. A simpler system with wider applications is the roller bottle reactors, which yielded around 2.5-fold higher glucose titers than shaken flasks with the same solids loading [164]. The authors found similar results when using bottles of different volumes between 125 mL and 2 L, which indicated the flexibility of the system.

The use of more adequate systems is also of utmost importance on a higher scale, resulting in effective mixing and improved mass transfer properties even at low mixing rates, reducing the energy consumption and improving the economic viability of the enzymatic hydrolysis at high-solids loading [160]. The usual Rushton turbine used in stirred tank reactors (STR) results in dead zones and only enables adequate mixing in the region near its paddles [160]. Therefore, different designs of both reactors and impellers have been proposed in an attempt to facilitate mass transfer and increase conversion yields.

One of the first changes in reactor design to allow for high-solids loadings was proposed by Mohagheghi et al. [20], who used a simple rotary fermenter for the simultaneous hydrolysis and fermentation of pretreated wheat straw with up to 24.4% solids concentration. A more recent study, by Jørgensen and coworkers [23], described and evaluated a modified reactor design that consisted of a free-fall horizontal drum with an internal mixing paddle. The horizontal configuration has the advantage of not being constrained by the solids loading, which is different from STRs, where there is a maximum initial load that allows the impeller to stir the mixture. This horizontal configuration allows the hydrolysis of a mixture with up to 40% (w/w) dry matter, yet the glucose yields obtained in this condition were merely above 30%.

The adaptation of the STR with more adequate paddles by substituting the Rushton turbine with a helical impeller was shown to be more adequate for mixing non-Newtonian fluids, such as the lignocellulosic biomass slurry, and enabled the hydrolysis at high-solids loading [160]. These authors observed that the use of the helical impeller allowed a significant decrease in energy consumption with a slight increase of 16% in the ethanol production in an SSF configuration from steam-exploded corn stover at 30% (w/w). Different impeller geometries were also studied by Battista and coworkers [165] for the hydrolysis of 20% (w/w) steam-exploded wheat straw using an STR. The use of a double-helical impeller resulted in a significant increase in glucose yields up to 64% in comparison to the anchor impeller and the paravisc impeller.

Du and coworkers [166] compared a horizontal bioreactor with an internal mixing blade to an STR with a double-helical impeller for the enzymatic hydrolysis of pretreated corn stover at 25% (w/w). The authors observed that the horizontal reactor allowed faster liquefaction of the slurry, which was attributed to better mixing properties, while the impeller in the STR could not stir in the first hours of hydrolysis due to the high viscosity of the mixture.

A novel mixing technology, called “periodic peristalsis,” that mimics the peristaltic movements in the stomachs of ruminants and requires less energy than the STR, was proposed by Liu and Chen [156]. These authors studied the hydrolysis of steam-exploded corn stover at solids loadings higher than 15% (w/w) and observed a slight hydrolysis yield improvement in comparison to shaken flasks of 12% at a solids loading of 27% (w/w), the highest tested. Viscosity results suggested that the “periodic peristalsis” improved the liquefaction rate in comparison to the experiments in shaken flasks. However, for solids loading higher than 24% (w/w), the liquefaction step lasted over 24 h, indicating a poor enzyme–substrate interaction due to mass transfer limitations.

Substrate characteristics should also be taken into account when choosing the more adequate reactor/impeller design. Ludwig and collaborators [117] proposed a segmented helical stirrer and evaluated this impeller design to improve the hydrolysis yields at solids loadings higher than 15% (w/w) of alkaline-pretreated wheat straw and organosolv-pretreated beechwood. The authors observed that the stirrer design was adequate for the hydrolysis of pretreated beechwood up to 30% (w/w) without significant mass transfer limitations. However, the system was only able to process 20% (w/w) pretreated wheat straw while already showing mass transfer limitations. Other works had similar findings when comparing the hydrolysis of different substrates in the same reactor configuration, indicating that the equipment design should be feedstock specific [115, 167].

Even though the use of different substrates and solids loadings hampers the comparison between the different reactor/impeller designs, some valuable observations can be made regarding the available data, which are summarized in Table 2. The helical impeller and the horizontal reactor, the most studied configurations, have been chosen as the most promising in comparison to other designs, allowing hydrolysis yields in the range of 60–70% regardless of the type of substrate or solids loading.

**Table 2:** Reactor and impeller designs proposed for the hydrolysis of lignocellulosic biomass at high-solids loadings in the range of 20–40%

Design of reactor/impeller	Solids loading (w/w) (%)	Substrate	Pretreatment type	Hydrolysis yield (%)	References
Shaking flasks	20	Wheat straw	Alkaline	30	[117]
Segmented helical impeller	20	Wheat straw	Alkaline	76	[117]
Horizontal reactor	20	Big bluestem	Hydrothermal followed by ultrasonic treatment	72	[169]
Horizontal reactor	20	Corncob residue	Dilute acid followed by alkaline treatment	63.8	[104]
Peg mixer	20	Unbleached hardwood kraft pulp	–	84	[168]
Periodic peristalsis	21	Corn stover	Steam explosion	71.2	[156]
Double helical impeller	25	Corn stover	Acid steam explosion	~ 60	[166]
Horizontal reactor	25	Corn stover	Acid steam explosion	~ 65	[166]
Horizontal reactor	25	Wheat straw	Steam explosion	~ 55	[23]
Segmented Helical impeller	30	Beechwood	Organosolv	72	[117]
Peg mixer	30	Agave bagasse	Organosolv	90	[163]
Horizontal reactor	40	Wheat straw	Steam explosion	~ 35	[23]
Paddle dryer	40	Wheat straw	Steam explosion	52	[120]

A different and promising impeller configuration, known as the “peg mixer” and commonly used in the pulp and paper industry, has been studied for the hydrolysis of unbleached hardwood kraft pulp and organosolv agave bagasse with high hydrolysis yields of 84% and 90%, respectively [163, 168]. These high yields, however, may be related to the use of materials more prone to enzymatic hydrolysis rather than to the mixing properties of this configuration. Thus, further studies with often-used substrates, such as straw, sugarcane bagasse, and corn stover, would be valuable to assess the true potential of the peg mixer configuration.

According to the reactor and impeller designs presented in Table 2, the diversity of substrates differently pretreated hinders a comprehensive comparison of the reported results regarding the best configuration. Thus, stepwise studies focusing on the hydrolysis results of a chosen substrate using different configurations of reactors and/or impellers, or the study of one proposed design for several substrates, would allow for more robust comparative data as well as to establish correlations between substrate characteristics and desired features of the reactor/impeller. The energy expenditure of the tested designs should also be evaluated, as this information is missing in most of the papers dealing with the topic and will be essential when evaluating the feasibility of the proposed technology.

The use of computational models can also be of help when designing new reactors and impellers. Gaona and co-workers [170] used computational fluid dynamics (CFD) to understand the mixing pattern of the tested impellers and how the insoluble solids distribution was affected by the mixing speed and impeller configuration. The first tested impeller resulted in a slow distribution of insoluble solids at lower mixing speeds. By analyzing the speed and volume fraction contours with CFD, the authors proposed a second impeller geometry, which proved to be more adequate for achieving a homogeneous suspension at lower mixing speeds and, therefore, with lower energy expenditure. The use of realistic systems with adequate mass transfer is thus necessary for better screening of the conditions needed for achieving yield improvements and cost reductions for the larger implementation of lignocellulosic conversion technologies in the industry.

## Research, technology, and commercialization based on high-solids enzymatic hydrolysis

The industrial compelling need for concentrated streams of sugars derived from the high-solids enzymatic hydrolysis of lignocellulosic materials, which is a mandatory part of the technology maturity (i.e., a requirement, not an option), has been pushing the research on different aspects aiming to face the challenges involved in the use of this renewable resource. However, the development of this technology on an industrial scale has been challenging, in spite of the substantial advances in this area.

**Fig. 1:**
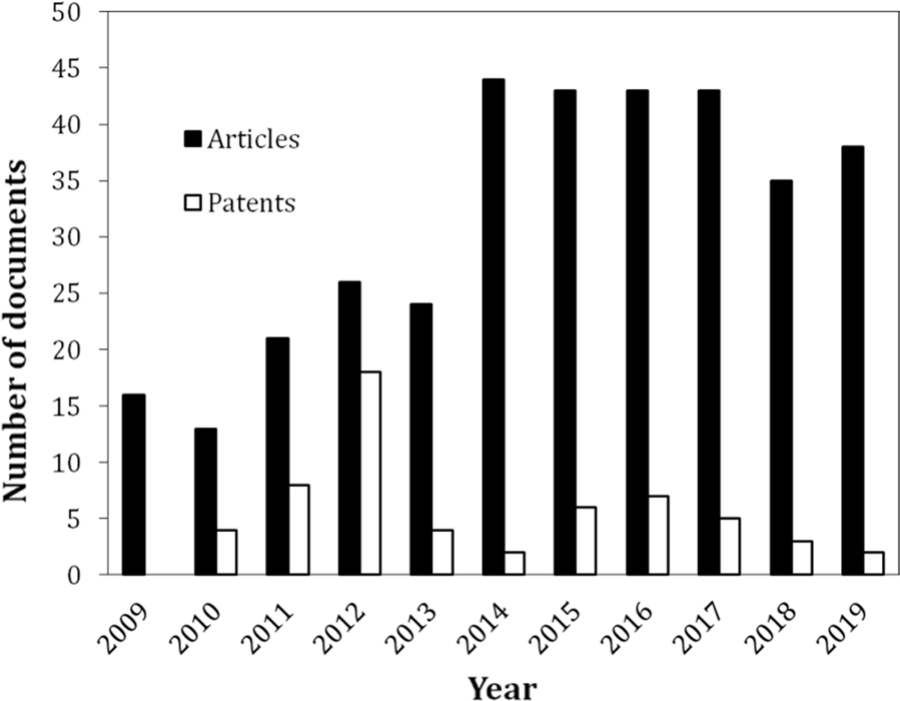
The number of papers and patents published from 2009 to 2019*. Strategy for the paper search on the Web of Science(346 documents): TS = (“high solids” AND “enzymatic hydrolysis”) OR TS = (“high solids” AND saccharification) OR TS = (“high consistency” AND “enzymatic hydrolysis”) OR TS = (“high consistency” AND saccharification) OR TI = (“high solid*” AND “enzymatic hydrolysis”) OR TI = (“high dry” AND “enzymatic hydrolysis”) OR TI = (“high solid*” AND saccharification) OR TI = (“high consistency” AND “enzymatic hydrolysis”) OR TI = (“high consistency” AND saccharification). Strategy for the patent search of the World Intellectual Property Organization (59 documents): EN_AB:(high NEAR solids AND enzymatic NEAR hydrolysis) OR EN_AB:(high NEAR solids AND saccharification) OR EN_AB:(high NEAR gravity AND enzymatic NEAR hydrolysis) OR EN_AB:(high NEAR gravity AND saccharification) OR EN_AB:(high NEAR consistency AND enzymatic NEAR hydrolysis) OR EN_AB:(high NEAR consistency AND saccharification) EN_TI:(high NEAR solid* AND enzymatic NEAR hydrolysis) OR EN_TI:(high NEAR solids AND hydrolysis) OR EN_TI:(high NEAR dry AND enzymatic NEAR hydrolysis) OR EN_TI:(high NEAR solid* AND saccharification) OR EN_TI:(high NEAR gravity AND enzymatic NEAR hydrolysis) OR EN_TI:(high NEAR gravity AND saccharification) OR EN_TI:(high NEAR consistency AND enzymatic NEAR hydrolysis) OR EN_TI:(high NEAR consistency AND saccharification) OR EN_TI:(high NEAR consistency AND hydrolysis NEAR cellulose) NOT EN_TI:(oxidized OR dietary OR treatment OR high-protein OR extraction OR lignin OR battery OR maltodextrin OR raw OR alkalinuria OR potato OR polyvinyl). *All searches were performed in November 2019

The progress of a given technology can be estimated by academic research papers that reflect the scientific interest, by the technological development linked to patent claims, and by the industrial activity. Advances in the high-solids hydrolysis developments will be discussed in the following to account for the academic, technological, and industrial interest. The results represent a search done using the Web of Science and World Intellectual Property Organization (WIPO) databases using the search strategies shown in Fig. 1.

The results of a survey regarding quantitative scientific publications and patents claimed are shown in Fig. 1. The description of the technologies addressed in the patents and the implementation of demonstration and commercial plants for cellulosic ethanol production are briefly discussed based on the data in Tables 3 and 4. Data collection for the overview of this technology was focused on the time frame from 2009 to 2019, which included a period of growing interest and significant investments in biofuels, mainly in the first 5 years. Later, some initiatives and investments decreased. Over 300 papers, with a prominent increase from 2014 onwards, have addressed the different subjects involved in high-solids hydrolysis, such as the type of biomass, the enzyme blend, the inhibitory effects, different reactor/impeller design, and the operation mode. The total number of patents, of 59, increased steadily from 2010 to 2012 and decreased noticeably from 2012 onwards, despite the also noticeable increase in scientific publications from 2013 until now. The reduction in investments in next-generation biofuels and biochemicals from 2012 onwards could be related to the decrease in the number of patents [10].

**Table 3:** Published patents recovered in the database of the World Intellectual Property Organization (WIPO) related to high-solids enzymatic hydrolysis

Patent title	Applicant/current assigned	Date of publication or grant^a^	Summary of the invention	References
Reactor for continuous saccharification of high-solids biomass	SK Innovation Co., LTD	2015	Design of a reactor for continuous saccharification of biomass at high-solids loading	[172]
High-solids biomass slurry generation for enhanced efficiency hydrolysis processing and equipment design to yield the same	Edeniq, INC.	2017	Shear cutting elements as impellers and a “pump ring” to enable high-solids enzymatic hydrolysis with high yields	[173]
Continuous countercurrent enzymatic hydrolysis of pretreated biomass at high-solids concentrations	Granbio Intellectual Property Holdings LLC	2018	Method of continuous countercurrent enzymatic hydrolysis in a reactor with perforated screw blades	[174]
Non-pressurized pretreatment, enzymatic hydrolysis and fermentation of waste fraction	Renescience AS	2018	High-solids enzymatic hydrolysis conducted in a free-fall mixing reactor	[175]
Enzymatic hydrolysis of cellulose	Borregaard AS	2012	Use of three hydrolysis tanks to conduct high-solids enzymatic hydrolysis	[176]
Process for improving the hydrolysis of cellulose in high-consistency systems using one or more unmixed and mixed hydrolysis reactors	Iogen Energy Corporation	2014	The use of an initial unmixed reactor and subsequently a mixed reactor	[177]
Enzymatic hydrolysis of biomasses having a high dry matter (DM) content	Inbicon A/S	2010	Design of a horizontal reactor for saccharification of biomass at high-solids loading	[178]
High-consistency enzymatic hydrolysis for the production of ethanol	Phillips Richard	2012	Thickening process by recycling the filtrate	[179]
Methods and systems for enzymatic hydrolysis of pretreated biomass at high-solids concentrations	Granbio Intellectual Property Holdings LLC	2019	Use of a surfactant and substrate recycling during high-solids enzymatic hydrolysis	[180]
Method for the hydrolysis of lignocellulosic biomass	Fiberight Limited	2018^b^	Use of a surfactant during high-solids enzymatic hydrolysis	[181]
Process for simultaneous saccharification and fermentation for production of ethanol	E. I. DuPont de Nemours and Company	2011	Simultaneous saccharification and fermentation at high-solids loading	[182]
High-solids enzymatic hydrolysis and fermentation of pretreated biomass	Abengoa Bioenergy New Technologies, LLC	2017	Temperature and dewatering control for high-solids enzymatic hydrolysis	[183]
Production of fermentable biomass sugars using high-solids enzymatic hydrolysis	API Intellectual Property Holdings, LLC	2015	Liquefaction and separation of solid and liquid streams to different uses	[184]
Enzymatic hydrolysis of pre-treated biomass	Andritz INC	2012	Whole method to conduct high-solids enzymatic hydrolysis	[185]
Methods of enabling enzymatic hydrolysis and fermentation of lignocellulosic biomass with pretreated feedstock following high-solids storage in the presence of enzymes	Catchlight Energy LLC	2012	The use of enzymes during storage and/or transportation of biomass at high-solids loading	[186]
Process for the rapid hydrolysis of high-solids biomass	Beta Renewables S.p.A.	2015	Continuous process of high-solids enzymatic hydrolysis	[187]

Search strategy: EN_AB:(high NEAR solids AND enzymatic NEAR hydrolysis) OR EN_AB:(high NEAR solids AND saccharification) OR EN_AB:(high NEAR gravity AND enzymatic NEAR hydrolysis) OR EN_AB:(high NEAR gravity AND saccharification) OR EN_AB:(high NEAR consistency AND enzymatic NEAR hydrolysis) OR EN_AB:(high NEAR consistency AND saccharification) EN_TI:(high NEAR solid* AND enzymatic NEAR hydrolysis) OR EN_TI:(high NEAR solids AND hydrolysis) OR EN_TI:(high NEAR dry AND enzymatic NEAR hydrolysis) OR EN_TI:(high NEAR solid* AND saccharification) OR EN_TI:(high NEAR gravity AND enzymatic NEAR hydrolysis) OR EN_TI:(high NEAR gravity AND saccharification) OR EN_TI:(high NEAR consistency AND enzymatic NEAR hydrolysis) OR EN_TI:(high NEAR consistency AND saccharification) OR EN_TI:(high NEAR consistency AND hydrolysis NEAR cellulose) NOT EN_TI:(oxidized OR dietary OR treatment OR high-protein OR extraction OR lignin OR battery OR maltodextrin OR raw OR alkalinuria OR potato OR polyvinyl)

^a^Date of publication of US Patent Office, except for the document, “Method for the hydrolysis of lignocellulosic biomass.”

^b^Date of publication of WO patent office

**Table 4:** The main demonstration and commercial plants of cellulosic ethanol

Company	Biomass	Country
Raízen	Sugarcane bagasse and straw	Brazil
GranBio	Sugarcane bagasse and straw	Brazil
Abengoa	Corn stover	USA
DuPont	Corn stover	USA
POET-DSM	Corn stover	USA
Inbicon	Wheat straw	Denmark
Clariant	Wheat straw	Romania
Beta renewables	Rice and wheat straw	Italy
Borregaard	Spruce	Norway

As expected, the number of patents related to high-solids enzymatic hydrolysis was lower than the number of papers (Fig. 1), as the scientific contribution could be relevant from TRL 1 to TRL 9 (technology readiness levels) and patents, in general, are claimed after experimental proof of the concept and a lab demonstration (TRL 3 and 4). Considering the processes described in the patents, the focus is mainly on the type of reactors/impellers and more general aspects of the parameters settled during high-solids enzymatic hydrolysis. It is also important to mention that the same invention is addressed in different patent documents to widen the patent protection in different countries (i.e., documents that are part of a patent family). Therefore, the total number of 59 documents found in the World Intellectual Property Organization database represented only 16 original technologies described in detail in Table 3.

The top subject of seven patents dealt with the design of new reactors and/or impellers [172–178], a strategy that has also been addressed in different scientific papers to overcome the hurdle of conducting high-solids enzymatic hydrolysis. As an example, the horizontal reactor proposed by Jørgensen et al. [23] is the subject of one patent that currently is assigned to the Inbicon S/A company [178]. That patent claims that a horizontal reactor with five chambers provides efficient mixing and improves the liquefaction stage even at 40% (w/w) solids loading. The inventors of the US Patent 8709770 [177] proposed the use of a reactor without agitation, named unmixed reactor, to diminish the viscosity of the media and subsequently complete the saccharification in a mixed reactor. Liu et al. [171], however, proposed an inverse approach using first a reactor with intensive mixing and then a saccharification reactor without agitation; their results indicated that this strategy resulted in major savings in power inputs.

Another important development subject to patent protection is related to the recycling of streams. The thickening process proposed by Phillips Richard [179] was studied by Geng et al. [145] and was found to result in a higher cellulose conversion by recycling the filtrate during high-solids enzymatic hydrolysis. The use of surfactants as additives to improve the enzymatic hydrolysis is also covered by several patents [180, 181]; this topic was based on studies by Knutsen and Liberatore [112], who observed that surfactants were a class of additives that provided better results for improving the rheological behavior of high-solids hydrolysis, which could be related to the lessening of the enzyme inhibition effect derived from the covalent binding of enzymes in lignin [147]. In a different approach, a DuPont patent refers to the simultaneous saccharification and fermentation process that uses enzymes alongside *Zymomonas mobilis* to obtain high titers of ethanol starting with a high-solids loading of biomass. Other aspects of the hydrolysis process (e.g., splitting of streams, temperature and pH control, the use of enzymes during storage and/or transportation of the biomass, and descriptions of continuous enzymatic hydrolysis methods) are claimed in documents listed in Table 3 as process strategies for the hydrolysis with high solids loadings.

Demonstrations and commercial plants are key indicators of the industrial interest in a given technology. Regarding biomass processing via biotechnological routes, several companies have constructed facilities in different countries where the biomass sugar syrups were used for the ultimate production of cellulosic ethanol (Table 4). Indeed, although enzymatic hydrolysis of lignocellulosic materials is a process that could precede the green production of several products, cellulosic ethanol plants achieved the commercial scale from a worldwide perspective [10]. Therefore, the cellulosic ethanol market has been the main driver to boost the development of the enzymatic hydrolysis of lignocellulosic biomass. Although it is often difficult to access the details of the processes implemented in the demonstrations and commercial plants due to industrial confidentiality, seven of the companies presented in Table 4 are related as applicants or current assigners of patents related to high-solids enzymatic hydrolysis. Although Raízen does not appear in Table 3, it is public knowledge that this company has implemented a process based on the Iogen Energy biofuel technology. Based on the patents evaluated, the need to increase the solids loading to improve the economic viability of lignocellulosic biorefinery was a goal during the last few decades by the main players in the cellulosic ethanol market.

Some of the listed companies have discontinued their operations for reasons more related to investments and public policies than to problems in enzymatic hydrolysis steps [10]. In addition, the growth in commercialization of cellulosic ethanol is a relevant parameter for the maturity of biomass processing via biotechnological routes. The Raízen Company, a joint venture between Shell and Cosan, has been reporting a growing production since 2016 and estimates the production of 2019 to be 16.5 million liters of ethanol [188]. The total capacity of the Costa Pinto Mill, which is located in the state of São Paulo in Brazil, is 40 million liters per year.

Both scientific and technological indicators are directly associated with private and public investments, and they are also subjected to fluctuations in economic boost and crisis periods, which includes oscillation in the market. A clear example is the period from 2000 to 2009, in which renewable energy technology experienced a global rise in interest. Governments, investors, and companies invested a record figure of US$147 billion in renewable energy (wind, solar, and biofuels). According to the 2019 report of Global Trends in Renewable Energy Investment, US$2.6 trillion were projected as investments in renewable capacity (excluding large hydro) in the last decade (2010–2019), with solar and wind leading the investments by accounting for almost 93% and biofuels representing approximately 0.9% of this total amount [189].

The research and technology related to high-solids enzymatic hydrolysis went through many ups and downs during the last decade following the pattern of global investment in next-generation biofuels and biochemicals [10]. The rise of more conservative ideas regarding environmental issues along with some frustrated initiatives related to the maturity of technology and its widespread use without considering the nature of biomass have resulted in a cooling down in the interest in this subject. In addition, solar and wind energy have gained more space and investments, as they are extremely important for the planet’s sustainability. However, it is important to remember how strategic this technology is for developing countries that generate large amounts of agro-industrial waste; moreover, its relevance is not only in the generation of biofuels but mainly in the implementation of a renewable-based chemical industry.

### Concluding remarks

A wealth of scientific and technical studies has meaningfully contributed to the maturity reached in the area of lignocellulosic materials’ conversion via enzymatic hydrolysis for the production of glucose. However, most of the work has been done using reaction mixtures with low-to-moderate solids loading, which do not suffer significantly with the “high-solids effect” (i.e., the decrease in cellulose conversion yields as solids loading increases). Therefore, there are still some gaps in knowledge that need addressing, which will help the successful establishment of the lignocellulosic industry.

A better understanding of the water-restrained environment of high solids loadings has been possible due to analytical tools that allowed the evaluation of the dynamics of water–biomass interactions and water constraint. Although there are indications that water constraint is a key factor that negatively affects high-solids enzymatic hydrolysis, it is also argued that the hindered mobility of free-flowing water imposed by the increased soluble species may be more relevant. These inconclusive findings could be resolved through analyses of the effect of water constraint using a broader spectrum of feedstock and pretreatments under comparative conditions. Knowledge regarding the contribution of different states and locations of water to the “high-solids effect” might also allow the development of process strategies to change the way water is constrained within biomass to improve the hydrolysis efficiency.

The water-restrained environment of high-solids enzymatic hydrolysis conveys rheological hindrances at the onset of the reaction and may strengthen the end-product inhibition of enzymes by glucose due to a reduced mass transfer coefficient. In addition, the high-solids milieu favors the increased concentration of molecular species that may hamper the enzymes’ performance, such as furans, phenols, cellobionic and gluconic acids, hemicellulose-derived sugars, and lignin, via wide-ranging mechanisms. In addition, the inefficient adsorption of cellulases to cellulose seems to be more pronounced at high solids loadings. Most of these inhibitory factors are dependent on the type of biomass, pretreatment, and the enzyme blend of choice and, therefore, could be somewhat mitigated by choosing the most suitable combination of these factors. Moreover, novel advances in enzyme formulations could alleviate such inhibitions, as was done in the past with the increase in β-glucosidase concentration to avoid cellobiose accumulation.

The scale-up of the high-solids enzymatic hydrolysis has been a challenging issue. Lignocellulosic slurries have non-Newtonian behavior and, therefore, require non-traditional reactors and impellers. Free-fall horizontal configurations and helical impellers are the most promising current technologies; in addition, the peg mixer configuration has shown interesting results but has been little explored. In any case, the extent of enzyme denaturation due to shear stress in each different reactor configuration would be worth evaluating. As the rheological characteristics of the biomass slurries are closely linked to the biomass type and the chosen pretreatment, a step-by-step evaluation of different pretreatments to a given biomass, and vice versa, would allow a more rational approach regarding the more advantageous duo biomass pretreatment. Nevertheless, the measurement of the rheological properties of lignocellulosic slurries is still a challenge due to the lack of specialized equipment. It has been indicated that the online measurement of the rheological properties would allow a better fitting of the mixing system as well as avoid sampling issues associated with such a heterogeneous mixture.

The fed-batch operation was shown to be an efficient strategy for the enzymatic hydrolysis of high total solids due to the significant advantage of maintaining a low viscosity medium. The choice of either the batch or the fed-batch process is not a minor one, as the main parameters (initial solids and periodicity of feeding) are interrelated with the chemical characteristics of the biomass, pretreatments, and enzymatic cocktail. Even though a fed-batch operation has been proven to maintain low viscosity, the effect does not directly translate in a higher sugar titer; fed-batch may, however, be advantageous in securing lower energy expenditures for mixing lower viscosity slurries.

Finally, techno-economic assessments are needed for choosing the most suitable combination of biomass, pretreatment, and enzyme preparation for high-solids hydrolyses, as most of the works aim to maximize glucose titers without accounting for the impact of the suggested adjustments on the cost of the final biomass sugar syrup.

## Data Availability

Not applicable.

## Abbreviations

AFEX: Ammonia fiber expansion
Aw: Water activity
CFD: Computational fluid dynamics
CMC: Carboximethycellulose
DM: Dry matter
FPU: Filter paper units
DP: Degree of polymerization
HBT: 1-Hydro-xybenzotriazole
5-HMF: 5-Hydroxymethylfurfural
LPMOs: Lytic polysaccharide monooxygenases
NMR: Nuclear magnetic resonance
PEG: Polyethylene glycol
SHF: Separate hydrolysis and fermentation
SSF: Simultaneous saccharification and fermentation
STR: Stirred tank reactors
SWO1: Swollenin
TRL: Technology readiness levels
WIPO: World Intellectual Property Organization
